# Ligand-modified multifunctional liposome-based targeted delivery platform: a multimodal cancer combination therapy strategy

**DOI:** 10.7150/thno.136152

**Published:** 2026-07-13

**Authors:** Xuehong Zhang, Yingjie Jiang, Xing Duan, Fuchun Li, Xiaozhuo Chen, Aikepaer Aikedai, Wanting Pan, Liangjie Ren, Yufei Su, Chengqi Li, Zhaoshuo Gao, Shengcai Liao, Qiang Zhang, Zhenyu Zhu, Kaipei Luo, Yingji Wang

**Affiliations:** 1 Department of Urology, Nanchang County People's Hospital. Nanchang 330200, Jiangxi, China.; 2 Chinese Medicine Germplasm Resources Innovation and Effective Uses Key Laboratory of Sichuan Province, School of Modern Chinese Medicine Industry, Chengdu University of Traditional Chinese Medicine, Chengdu, 611137, China.; 3 Laboratory of Aging Research and Cancer Drug Target, State Key Laboratory of Biotherapy and Cancer Center, National Clinical Research Center for Geriatrics, West China Hospital, Sichuan University, Chengdu 610041, China.; 4 Department of Breast Surgery, Kyoto University Graduate School of Medicine, Kyoto, 606-8507, Japan.; 5 Department of Geriatric medical center, Sichuan Provincial People's Hospital, University of Electronic Science and Technology of China, Chengdu, China.; 6 Chengdu Women's and Children's Central Hospital, School of Medicine, University of Electronic Science and Technology of China, Chengdu, 611731, China.; 7 College of Pediatrics, Chongqing Medical University, Chongqing, China.; 8 Department of Pharmacy, Personalized Drug Therapy Key Laboratory of Sichuan Province, Sichuan Academy of Medical Sciences & Sichuan Provincial People's Hospital, School of Medicine, University of Electronic Science and Technology, Chengdu 610054, China.; 9 Department of Critical Care Medicine, Frontiers Science Center for Disease-related Molecular Network, State Key Laboratory of Biotherapy and Cancer Center, West China Hospital, Sichuan University, Chengdu 610041, China.

**Keywords:** ligand modification, multifunctional liposomes, targeted delivery, cancer combination therapy

## Abstract

The combination therapies are significantly more effective than monotherapies in enhancing anticancer efficacy, reducing drug-related toxicity, and lowering the risk of drug resistance in cancer treatment. However, achieving precise delivery of the drugs to the tumor site remains a major challenge. With the deepening exploration of surface-engineered nanocarriers, ligand-modified liposomal drug delivery systems (LLDDS) are constructed by integrating the active-targeting properties of functional ligands (such as peptides, glycans, and aptamers) with the inherent advantages of liposomes. LLDDS show promise for exhibiting strong tumor-targeting capability, improving pharmacokinetics, biodistribution, and therapeutic efficacy of anticancer agents, such as chemotherapy drugs, and enabling the multifunctional integration of multiple therapeutic strategies. This review summarizes the development of liposomes and ligand-mediated surface modification strategies. More significantly, the development of multifunctional liposomes, targeted delivery, improved anticancer effectiveness, and possible anticancer mechanisms are highlighted in the discussion of LLDDS's recent advancements for integrated therapy approaches in a variety of malignancies. Lastly, the potential and difficulties of clinical translation in this ever-evolving area are examined.

## 1. Introduction

To date, cancer remains a major global public health challenge threatening human health 1. With advances in medical technology, novel approaches for cancer treatment have emerged. These include chimeric antigen receptor T-cell (CAR-T) therapy and immune checkpoint blockade (ICB) therapy, in addition to surgical resection, chemotherapy, and radiotherapy 2, 3. However, in clinical practice, chemotherapy is still the most fundamental and widely used treatment modality. Unfortunately, chemotherapy not only kills tumor cells but also affects the body's normal cells, leading to severe side effects. Additionally, low solubility, a short circulation half-life, and multidrug resistance restrict the administration of traditional chemotherapy drugs 4-6. Addressing these challenges requires precise control over drug distribution and concentration *in vivo* to improve delivery efficiency to tumor sites. In the meanwhile, combination treatment is gradually replacing monotherapy in cancer research. All the strategies can have their strengths enhanced by combining them, and individual deficiencies may also be addressed 7, 8.

To address the problems of clinical application for chemotherapeutic drugs, a large number of new drug-delivery methods have been developed in recent years. Targeted drug delivery systems (TDDS) are designed to deliver drugs to specific sites in the body precisely, and have shown good results so far 9, 10. TDDS can be broadly divided into passive targeting and active targeting based on receptor-ligand interactions. Passive targeting primarily depends on the enhanced permeability and retention (EPR) effect, which has been observed in many solid tumors 11. The EPR effect was first observed by Matsumura and Maeda in 1986 12. It refers to the enhanced accumulation of nanoparticles (NPs) in tumors through abnormal and leaky vasculature, followed by retention due to impaired lymphatic drainage. Nanodrugs with particle sizes ranging from 10 to 100 nm can preferentially accumulate in tumor tissues rather than in normal tissues 13, 14. However, preclinical studies have yielded inconsistent results. Some researchers have suggested that the EPR effect shows substantial heterogeneity across tumor types, among patients, and even within different regions of the same tumor 15, 16. Furthermore, the EPR effect is usually more uniform in small and rapidly growing tumors. As a result, the EPR effect in small animal tumor models is expected to differ significantly from that in humans, and its reliability is therefore limited 17, 18. To sum up, these issues have prompted researchers to re-evaluate the extent to which drug delivery depends on the EPR effect, a concept that remains highly controversial. On the other hand, ligands that identify and attach to proteins, lipids, or carbohydrates on the surface of tumor cells that are overexpressed or selectively expressed under pathological circumstances are used in active targeted delivery 19, 20.

In the design of actively targeted anticancer nanodrug delivery systems, liposomes offer substantial advantages, including excellent biocompatibility, reduced systemic toxicity, improved drug stability, and prolonged circulation half-life 21. Several liposomal nanomedicines have been approved for clinical use, such as Doxil, DaunoXome, Myocet and Vyxeos. Compared to traditional liposomes lacking intrinsic active targeting mechanisms, ligand-modified liposomes demonstrate enhanced tumor accumulation and more specific cellular uptake, thereby significantly improving anticancer efficacy 22, 23. Common targeting ligands include peptides (e.g., cell-penetrating peptides and cell-targeting peptides), glycans (e.g., mannose, fucose, and chondroitin sulfate), aptamers (e.g., AS1411 and PtdSer), folic acid, transferrin, and nanobodies. More importantly, by loading drugs, ligand-modified liposomes can serve as multifunctional drug delivery platforms that integrate multiple cancer therapies, enhance their targeting capability, and ultimately achieve robust and comprehensive therapeutic effects 24, 25. Emerging treatment approaches, including as immunotherapy, photothermal therapy (PTT), photodynamic therapy (PDT), chemodynamic therapy (CDT), and ferroptosis-based therapy, have been thoroughly studied in addition to traditional chemotherapy and radiation. Numerous *in vitro* and *in vivo* investigations have shown the synergistic therapeutic benefits of various approaches.

**Figure 1:**
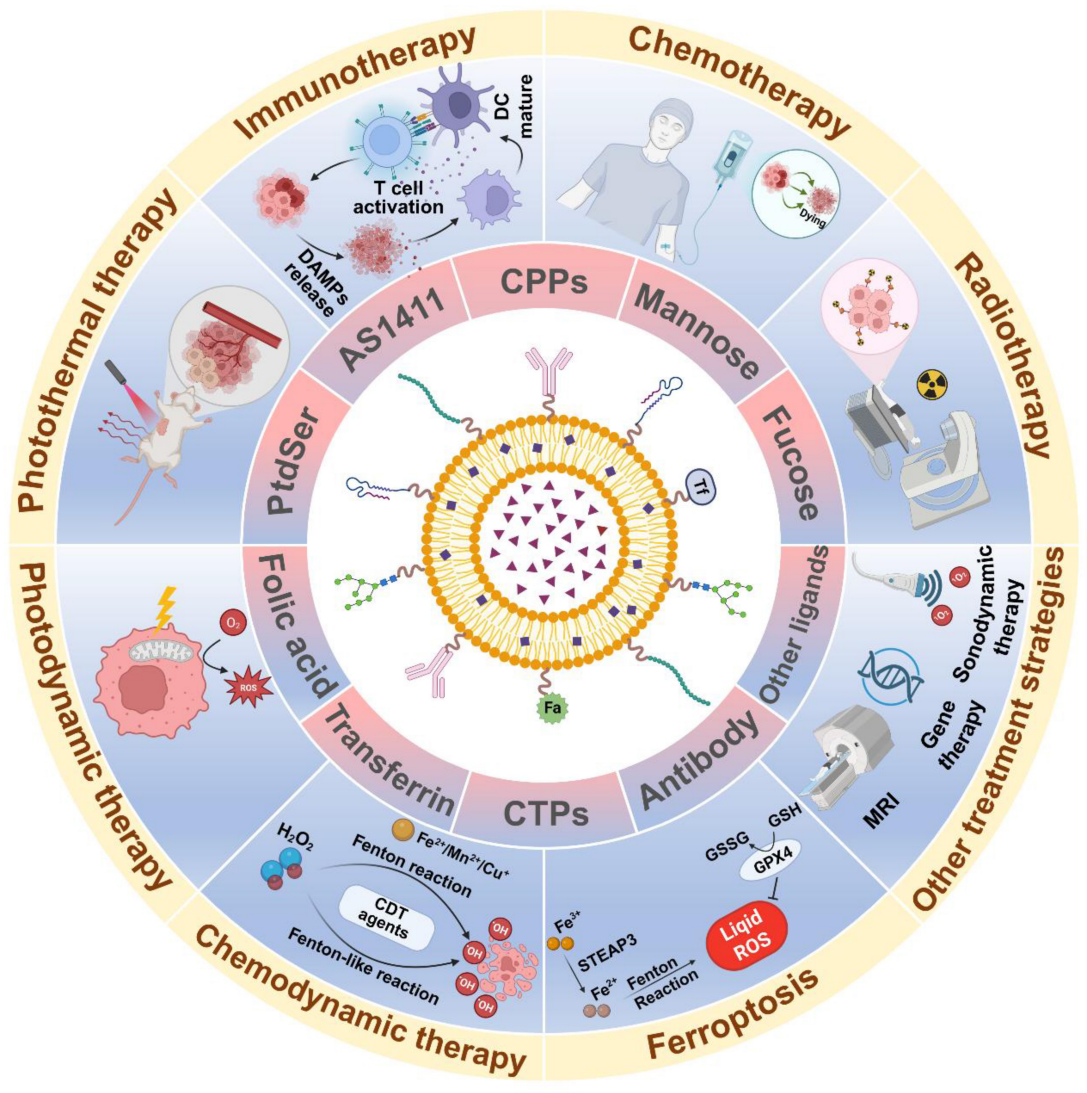
Scheme of ligand-modified liposomal drug delivery systems for combination cancer therapy. Liposomal nanoplatforms functionalized with diverse ligands, including peptides such as CTPs and CPPs, glycans such as mannose and fucose, aptamers such as AS1411, antibodies, folic acid, and transferrin, can enhance tumor accumulation, promote receptor-mediated cellular uptake, and enable controlled drug delivery. By co-delivering therapeutic agents and functional components, these ligand-modified liposomes can integrate chemotherapy, immunotherapy, radiotherapy, photothermal therapy, photodynamic therapy, chemodynamic therapy, sonodynamic therapy, gene therapy, ferroptosis-based therapy, and imaging-guided interventions. The multifunctional ligand-modified liposomal drug delivery systems provide a versatile platform for precise and synergistic cancer treatment.

Herein, we summarize the recent developments in ligand-modified liposomal drug delivery systems (LLDDS) for multimodal cancer therapy (**Figure 1**). First, we outline the evolution of liposomal systems, from first-generation conventional liposomes to second-generation polyethylene glycol (PEG)-modified liposomes and, ultimately, third-generation ligand-modified liposomes. These three generations' benefits and drawbacks are contrasted, with special attention paid to how well ligand-modified liposomes work in comparison to regular liposomes. Next, the main strategies for conjugating targeting ligands to liposomes are introduced. More remarkably, we have systematically investigated integrated strategies for applying ligand-modified liposomal nanotechnology to combination cancer therapy, with a focus on liposomal formulation, targeted delivery, controlled drug release, and anticancer activity. Lastly, we go over the main obstacles and potential opportunities related to the practical use of LLDDS for multimodal combination cancer treatment. This study seeks to further the development of customized, selective, and multifunctional cancer therapeutics as well as contribute to a paradigm shift in precision nanomedicine by a thorough examination of several active targeting ligands and liposomal delivery methods.

## 2. Materials and methods

We searched English databases between 2023 and 2026, including PubMed, Web of Science, and Google Scholar, then we screened relevant literature published in China and abroad. The databases were searched using the following terms: [‘‘ligand” OR ‘‘peptide” OR ‘‘aptamer” OR ‘‘glycan” AND ‘‘liposome”]. Liposomal drug delivery systems were retrieved in the database using the following terms: [‘‘liposome” AND ‘‘cancer”]. According to the situation of different databases, the subject words are comprehensively searched in combination with keywords, topics, abstracts, and free words to ensure the systematization and integrity of literature retrieval.

We searched all preclinical studies on the ligand-modified liposomal drug delivery systems for combination cancer therapy. To ensure the authenticity and systematicness of the results, we included relevant *in vitro* and *in vivo* studies involving cell and animal models.

## 3. The development process of liposomes

First-generation liposomes are those with simple lipid molecular structures. In 1965, British researchers Bangham and Standish discovered first-generation liposomes by dispersing phospholipids in water for electron microscopy observation. When phospholipids are dispersed in water, they naturally form multilayer vesicles, with each layer consisting of a lipid bilayer. The central region of the vesicles, as well as the spaces between layers, is filled with water, and the bilayer thickness is approximately 4 nm. In 1971, Rymen *et al*. from the UK utilized liposomes for drug delivery by encapsulating hydrophilic compounds in the aqueous core and binding hydrophobic compounds to the lipid bilayers. The simultaneous encapsulation of hydrophilic and hydrophobic medications, as well as their degradability and biocompatibility, were among the many benefits offered by first-generation liposomes. However, they also faced challenges, such as instability, low hydrophobic drug loading capacity, limited release of hydrophilic drugs, and a short blood half-life 26.

To correct the deficiencies of the first generation of liposomes, second-generation liposomal systems have been developed with improved *in vivo* stability and extended circulation time. Traditional liposomes are easily recognised, taken up and cleared by the mononuclear phagocyte system after intravenous injection, and thus have a limited therapeutic effect; therefore, these modified formulations have reduced rapid immune clearance and improved their prospects for clinical application. Blume and Klibanov modified the surface of liposomes with PEG in 1990 to address this problem and developed PEGylated liposomes. PEGylated doxorubicin liposomes (Doxil®) in 1995 were the first PEGylated nanomedicines approved by the US Food and Drug Administration and performed well. Doxil® was a precursor; now, several PEGylated liposomes, such as ThermoDox® and MM-302, have also advanced to clinical trials 27-28. PEGylation alters the protein corona on the surface of liposomes to extend their circulation time in the blood and enhance drug delivery. PEG chains introduce steric hindrance at the surface of liposomes and, in turn, prevent aggregation caused by van der Waals forces. Thus, there will be no aggregation in the formulation. PEGylation can be added to liposomes to give them a "stealth" effect and thus prolong their circulation in the body. PEGylated liposomes take advantage of the EPR effect in tumours to prolong their circulation time and thus accumulate more drugs at the tumour site passively 29. However, PEG modification has defects such as reduced drug uptake and impaired endosomal escape. Recently, some studies have shown that repeated administration of PEGylated liposomes induces the generation of anti-PEG antibodies. Antibodies bind to PEGylated liposomes, activate the complement system and increase the risk of allergic reactions 30.

**Figure 2:**
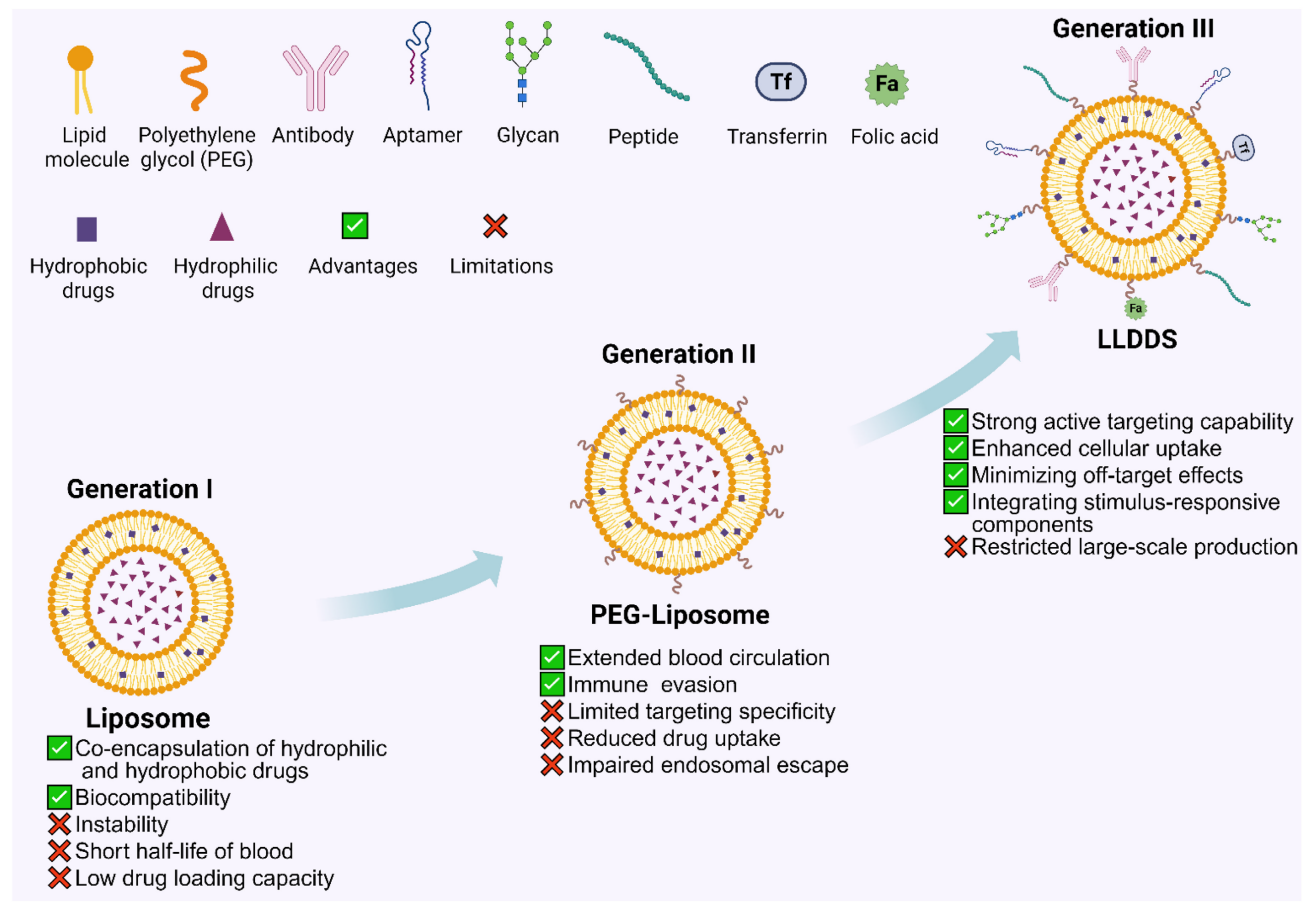
The development process of liposomes: from first-generation conventional liposomes to second-generation PEG-modified liposomes and, ultimately, third-generation ligand-modified liposomal drug delivery systems (LLDDS). The advantages and limitations of each generation are shown in the diagram of the respective liposomes.

Third-generation liposomes were developed to overcome the deficiencies of second-generation PEG liposomes, such as the protein corona on their surfaces that prevents cell entry. EPR effect is not beneficial to the therapeutic effect of nucleic acid, protein and peptide drugs that require good cell permeability. Given the above problems, many scholars have carried out relevant studies and developed third-generation liposomes. Liposomes are equipped with ligands, such as peptides, glycans, aptamers and antibodies, in a particular way. Due to the fluidity of lipids, surface-bound ligands have relatively more freedom in the lipid bilayer and are therefore better able to bind to receptors and pass through cells 31. Ligand-modified liposomes improve the efficacy of drug delivery systems by selectively targeting and binding receptors overexpressed on tumor cell surfaces, while sparing healthy cells. Furthermore, minimizing off-target effects and integrating stimulus-responsive components are key advantages of third-generation liposomes. One of the major challenges in applying third-generation liposomes is scaling up. The absence of engineering advancements and accurate mathematical models to understand these processes hinders large-scale production (**Figure 2**).

## 4. Ligand modification strategies of functionalized liposomes

In the construction of multifunctional liposomes based on ligand modification, two primary approaches are employed. The first approach involves directly mixing phospholipids with functional elements or active functional groups together with other phospholipid cofactors. This process enables the target moiety to be attached to the lipid. The second method is to functionalize the preformed liposomes with the desired target ligand on the liposome surface.

In both of the aforementioned methods for constructing functionalized liposomes, surface modification is achieved through the formation of covalent bonds. By creating strong chemical interactions, covalent bonding directly affixes functional components—like peptides, aptamers, or antibodies—to the liposome surface. Common covalent bonding methods include amide bond, hydrazone bond, and thioester bond. For example, aptamers or antibodies can be covalently attached to liposomes using these methods 32. The first is that these strategies have high stability and, therefore, the functionalized liposomes are effective for a longer time. In addition, this way can control the concentration and distribution of functionalised liposomes more precisely to improve the efficiency of targeted drug delivery. Although covalent bonds are very stable, their synthesis involves numerous chemical reactions and relatively complex steps; thus, they are difficult to prepare in a lab. Due to the relatively complex synthesis process, the cost of raw materials and equipment is also high 33-34. Non-covalent bonds are used to connect ligands with liposomes by means of physical and mechanical forces. Common non-covalent bonding modes are electrostatic interactions, hydrophobic interactions and simple adsorption. Cationic liposomes can bind negatively charged nucleic acids or peptides through electrostatic attraction, for example 35. Non-covalent bonding is relatively simple, low in production cost and easy to realise; therefore, it is suitable for large-scale production. However, the bonded type is unstable in the presence of serum components, enzymes or pH changes *in vivo* and is therefore prone to degradation. These factors may cause ligand dissociation and thus reduce the drug's targeting and controlled-release effects. In addition, non-covalent bonds are relatively weak physical forces; thus, they may not effectively stabilize the liposome 36.

## 5. Ligand-modified liposomal drug delivery systems for cancer combination therapy

### 5.1. Peptide

Peptides are necessary active substances in the body, and they consist of oligomers of amino acids linked by amide bonds. Peptides have the advantages of high specificity, strong affinity and targeting capabilities. Peptides are also able to cross cell membranes and are responsive to the environment; thus, they have many applications in diagnostics and targeted drug delivery 37. Research has shown that the biological activity of peptides is closely related to their conformation, such as α-helices or β-structures, which promote amphipathic behaviour and are necessary for their biological functions 38. The two kinds of peptides are cell-penetrating peptides (CPPs) and cell-targeting peptides (CTPs). CPPs have up to 35 amino acids and are more biocompatible, more permeable to membranes, less toxic and less immunogenic than other cationic polymers. CPPs have shown promising results as modification ligands due to their excellent tissue penetration ability, and thus can achieve targeted drug delivery in a non-destructive manner. Polyarginine is a typical CPP that has been used to deliver therapeutic agents. R8 is an insulin delivery device and R7 is a cyclosporine A delivery device, for example 39, 40. CTPs are short peptides that have a high affinity and specificity for cellular or tissue targets. CTPs can bind to specific molecules on the surface of target cells, such as receptors and glycoproteins, to deliver drugs, genes and other therapeutic substances into the target cells 41. Peptides bind to cell-surface receptors, are taken up via endocytosis, and then both the peptide and its therapeutic payload enter the cell 42. This strategy allows CTPs to improve the targeted transport and intracellular delivery of therapeutic agents, including drugs and genetic materials, while reducing off-target toxicity in normal or non-targeted cells 29, 43. Peptides can be easily chemically synthesized, modified, and conjugated to liposomes through various methods. The most common chemical linkages include disulfide and thioester bonds, which covalently attach peptides to liposomes. Reactive functional groups in peptides, such as -COOH, -NH₂, and -SH, provide ideal sites for conjugation with liposomes, especially with DSPE-PEG-Mal/NHS 44, 45. Interestingly, in tumor therapy, peptide ligands enhance tumor penetration and reduce the MPS-mediated clearance of liposomes. Research on using peptide-modified liposomes to deliver drugs to cancer cells is gaining increasing attention, particularly with the growing interest in cancer combination therapies.

#### 5.1.1. Peptide-modified liposomes enabling chemo-immunotherapy synergy

Chemotherapy is a central modality in cancer treatment. It directly kills cancer cells and induces immunogenic cell death (ICD), playing a crucial role in enhancing therapeutic efficacy in multimodal treatment strategies. ICD is a specialized form of apoptosis that activates cellular immunity and restores anticancer immune responses 46. During ICD, cancer cells release damage-associated molecular patterns (DAMPs), such as calreticulin (CRT), adenosine triphosphate (ATP), and high-mobility group box protein 1 (HMGB1) 47, 48. DAMPs can bind to and promote the maturation of dendritic cells (DCs). DCs present antigens to cytotoxic T lymphocytes, activating T cell-mediated immune responses. Nevertheless, chemotherapeutic intervention may unintentionally induce an immunosuppressive tumor microenvironment, thereby weakening antitumor immune activity and reducing overall therapeutic effectiveness. Chemo-immunotherapy, which combines chemotherapy with immunotherapy, has emerged as a breakthrough strategy for targeting and eliminating malignant tumors, fundamentally transforming cancer treatment 49. Significantly, peptide-functionalized liposomal drug delivery systems have significantly advanced chemo-immunotherapy by synergistically delivering both chemotherapeutic and immunotherapeutic agents, while also modulating the TME.

RGD is a well-known peptide sequence that targets tumors by binding to integrin receptors, namely αvβ3 and αvβ5 integrins, on the surface of tumor cells. These integrin receptors are frequently upregulated in malignant cells and tumor-associated vascular endothelial cells, which makes RGD peptides highly suitable targeting ligands for tumor-directed therapeutic delivery. RGD peptides not only promote endocytosis of tumor cells through integrin binding but also enhance the localization and delivery of anticancer drugs, thereby improving therapeutic efficacy 50. In addition, cRGD peptides reduce the flexibility and instability inherent in free-chain peptides by cyclizing the RGD sequence, thereby enhancing their affinity and selectivity for integrin receptors. The cyclic structure enhances their stability *in vivo*, improving their performance in targeting tumor cells and tumor vasculature. cRGD peptides are widely used to target tumor cells and tumor-associated angiogenesis 51. Wang *et al*. 52 proposed a strategy to degrade signal transducer and activator of transcription 3 (STAT3) using nano-integrated proteolysis-targeting chimeras (PROTACs). This approach efficiently reprogrammed hepatocellular carcinoma (HCC)-associated cancer stem cells (CSCs), suppressed the growth of CSCs, and stimulated anti-HCC immune responses. PROTAC technology is an innovative and promising therapeutic strategy that uses small molecules to induce ubiquitin-dependent protein degradation. PROTACs are developed by linking the target protein (POI) to ligands and E3 ligase ligands via an intermediate linker. inS3-TEG-VL-TK was incorporated into cRGD-modified cationic liposomes to enhance uptake by CSCs that overexpress integrin αvβ3, facilitating lysosomal escape and promoting interaction with cytoplasmic STAT3. The liposome (Lip@inS3-TEG-VL-TK) efficiently depleted endogenous STAT3 in CSCs, suppressed their stemness, induced anticancer immunity, and protected healthy cells, thereby reversing HCC progression. Furthermore, fluorescence imaging showed that the cRGD-modified liposome evaded hepatic and renal clearance. The cRGD-modified liposome also exhibited significantly higher tumor-specific accumulation than the non-RGD-modified liposome. This further supported the tumor-targeting efficacy of the liposomal PROTAC prodrug *in vivo* and validated the need for the cRGD-modified liposomal carrier. Relative to the control cohort, in which the mean number of metastatic nodules reached 21, treatment with Lip@inS3-TEG-VL-TK markedly inhibited pulmonary dissemination of HCC, lowering the average nodule burden to 1. This study presented a modular therapeutic strategy using peptide-modified liposomes to enhance the efficacy of HCC chemotherapy-immunotherapy combinations.

Previous studies have demonstrated that stimulation of the aryl hydrocarbon receptor (AhR) in splenic dendritic cells downregulates the production of key cytokines. These cytokines are critical for T helper cell polarization. A ligand with strong affinity and specificity for DCs was created by Zhang *et al*. 53, named synthetic peptide 65 (SP65), which was identified through phage display as a DC-targeting peptide. SP65 was conjugated to the neutral lipid dioleoylphosphatidylethanolamine (DOPE) via a linker to form SP65-DOPE. The phospholipid-peptide complex was incorporated into the surface of liposomes, forming SP65-lipo-CH. Uptake experiments showed that SP65-lipo-DiR treatment increased DiR intensity by 1.6-fold in the spleen and 1.8-fold in the kidneys compared with the non-SP65-modified group. Interestingly, SP65-lipo-CH significantly increased IL-12 production, activated NK cells and CD8^+^ T cells, and elevated interferon-γ (IFN-γ) levels. These changes led to effective anticancer activity against MC38 colorectal cancer and LLC lung cancer in mice. Similarly, Zhang *et al*. 54 designed a three-step artificial intelligence (AI) workflow to accelerate the design of dual-drug-loaded lipid carriers targeting CXCR4 for colorectal cancer treatment. Flexible docking and AlphaFold-based interface scoring identified a 9-mer fragment of SDF-1 (pSDF-1), known as the CXCR4-binding peptide (CXCR4BP). The CXCR4BP-modified liposome (with PTX located in the lipid shell) encapsulated berberine (BBR)-loaded functionalized mesoporous silica nanoparticles (FMSN), forming FMSN(BBR)-CXCR4BPL(PTX) for synergistic chemo-immunotherapy in colorectal cancer. The platform demonstrated high dual-drug encapsulation efficiency (78.8 ± 1.9% for BBR and 75.2 ± 2.4% for PTX), with sustained drug release over 72 hours. Concurrently, *in vivo* imaging confirmed that CXCR4BP conferred robust and selective targeting of CXCR4-positive CT26 colorectal cancer cells on the liposome. CXCR4BP modification also enhanced tumor accumulation without detectable uptake in non-target organs. *In vivo* anticancer studies showed that FMSN(BBR)-CXCR4BPL(PTX) caused significant tumor regression, reversed splenomegaly, and exhibited potent anti-proliferative, pro-apoptotic, and anti-angiogenic effects.

Moreover, octaarginine (R8) is a short peptide consisting of eight arginine residues. Due to its positive charge, the R8 peptide interacts with the negative charges on cell membranes, facilitating its transport across the membrane. R8 peptides are frequently used as CPPs to efficiently deliver bioactive molecules, such as small-molecule drugs, DNA, and RNA, into cells 55. R8-modified liposomes (R8-Lip) were shown to enhance antigen presentation by MHC class I (MHC-I) in dendritic cells after antigen encapsulation. Nakamura and others 56 have shown that encapsulating polyinosinic-polycytidylic acid and ovalbumin (OVA) in R8-Lip significantly enhances their efficacy after intravenous injection. Treatment with the R8-Lip/PIC/OVA system increased the immune status of B16-OVA tumours and converted them from "cold" to "hot" tumours.

#### 5.1.2. Peptide-modified liposomes enabling photo-chemotherapy synergy

Phototherapy has developed rapidly in recent years and now uses light of a particular wavelength to induce photochemical changes or heat damage in cancer cells and tissues. Among the various kinds of light, Photodynamic Therapy (PDT) and Photothermal Therapy (PTT) have been extensively studied and applied to date 57, 58. Photodynamic therapy is the irradiation of a specified wavelength to activate photosensitizing agents that have accumulated in diseased tissues, generate cytotoxic reactive oxygen species (ROS), particularly singlet oxygen, and thus cause localised damage to the target tissues 59. Photothermal therapy is a treatment modality in which absorbed light is converted into thermal energy. Upon photoexcitation, photothermal agents dissipate the absorbed energy through non-radiative relaxation as they return to the ground state, thereby producing heat and elevating the temperature within the local microenvironment. This localized hyperthermia can damage or eliminate tumor cells and pathogenic bacteria, while also inducing multiple biological alterations in cancer cells 60, 61. Owing to their different modes of action, photodynamic therapy and photothermal therapy can serve as effective complements to conventional chemotherapy. At the cellular level, these light-based therapies may help reverse chemoresistance and restore therapeutic sensitivity by influencing dysregulated signaling networks. Within the tumor microenvironment, PDT and PTT can further improve tumor perfusion, increase vascular permeability, and remodel extracellular matrix barriers, thereby facilitating more efficient intratumoral drug accumulation and penetration. Furthermore, compared to chemotherapy-mediated systemic therapies, PDT and PTT offer the advantage of local specificity, providing better spatio-temporal control and reducing off-target toxicity 62, 63. In addition to encouraging neutrophil recruitment and complement cascade activation, PDT and PTT may simultaneously increase the release of immune-modulatory cytokines including IL-6 and interleukin-4 (IL-4). Together, these responses establish an acute inflammatory milieu that supports the infiltration and activation of immune cells. Importantly, ICD triggered by PDT or PTT is associated with the liberation of DAMPs, which function as endogenous alarm signals to initiate and amplify antitumor immune responses 64, 65. Combined chemotherapy and phototherapy can synergistically activate the immune system, enhancing therapeutic efficacy, particularly when combined with immunotherapies like immune checkpoint inhibitors. Integrating chemotherapy and phototherapy into a combined photo-chemotherapy strategy aims to overcome the limitations of monotherapy by leveraging the complementary effects of both approaches. The precision of phototherapy, the systemic action of chemotherapy, enhanced immune responses, and the ability to overcome drug resistance have generated growing interest in photo-chemotherapy.

**Figure 3:**
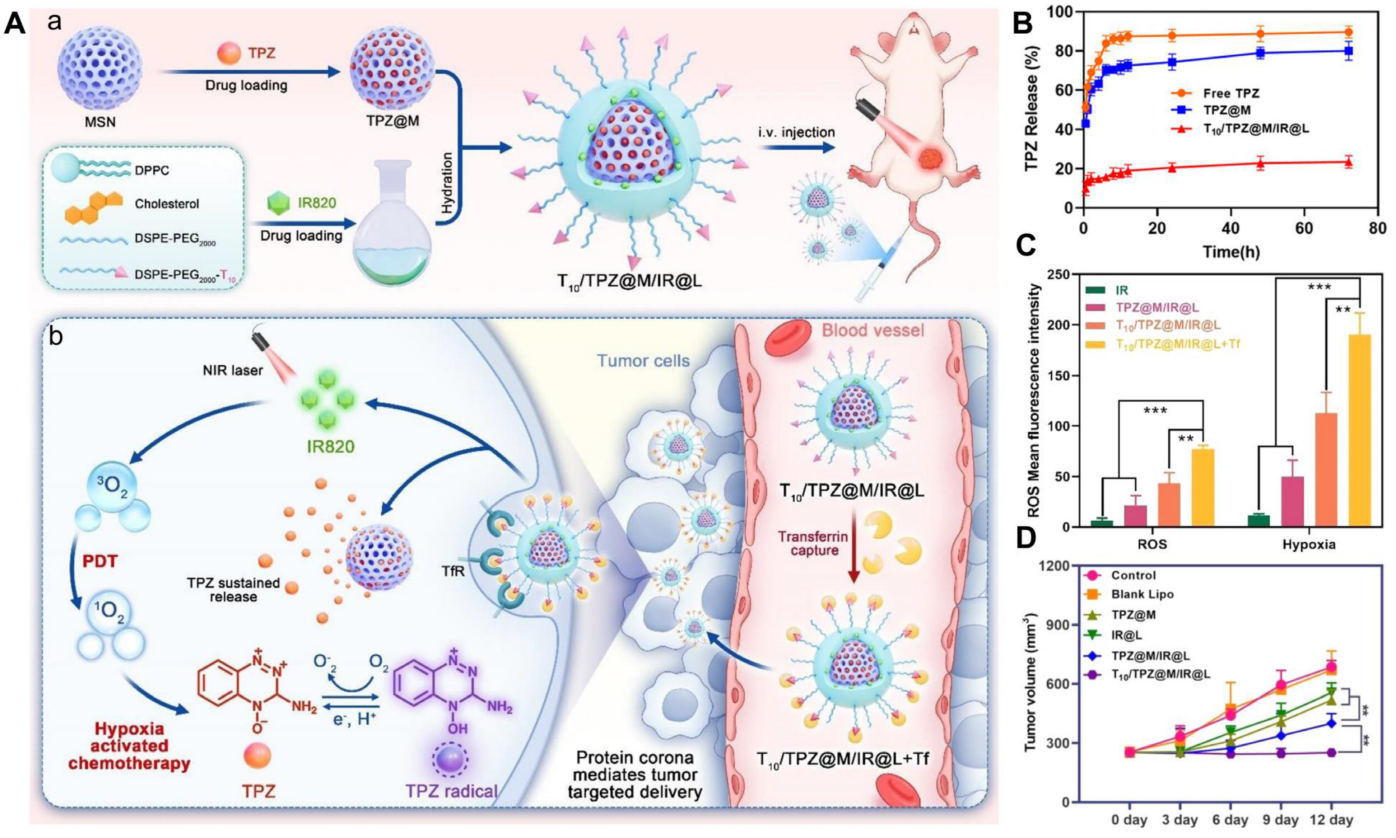
Peptide-modified liposomal drug delivery systems for cancer photo-chemotherapy. (A): (a) Schematic illustration of design and preparation of the T_10_/TPZ@M/IR@L and (b) hypoxia-induced chemo-phototherapeutic effect under the mediation of Tf protein corona. (B) *In vitro* drug release of TPZ. (C) *In vitro* detection of reactive oxygen species/hypoxia in 4T1 cells treated with different formulations. (808 nm, 100 W/cm^2^, 5 min). (D) Tumor growth volume of the mice after different treatments. Adapted with permission from 67, Copyright 2024, American Chemical Society.

Recent studies have shown that when nanomaterials are introduced into biological systems or fluids, proteins in the bloodstream spontaneously adsorb onto nanoparticle surfaces, forming a “protein corona” (PC) 66. While PC confers novel biological properties to nanoparticles, it impairs their functionality and targeting within cells. As a result, researchers are exploring the use of adsorbable proteins with active ligand properties to modify nanoparticle surfaces, forming endogenous PC *in situ*. This allows the ligand proteins to guide the nanoparticles to target sites. For example, Jin *et al*. 67 used the T_10_ peptide (sequence: CGGGHKYLRW), which has a high affinity for transferrin (Tf) and selectively binds Tf in the body. Transferrin (Tf) is abundant in the bloodstream, whereas its receptor, the transmembrane protein transferrin receptor (TfR), is frequently upregulated in diverse malignant cells. This expression pattern makes the Tf/TfR axis an attractive strategy for pre-PC tumor-targeted delivery. This study used T_10_ peptide-modified liposomes to create *in situ* Tf-PC-mediated liposomes carrying the hypoxia-sensitive chemotherapy drug tirapazamine (TPZ) and the photosensitizer indocyanine green (IR820) (**Figure 3A**). The water-soluble drug TPZ was encapsulated in mesoporous silica nanoparticles (MSNs) and coated with IR820-loaded liposomes (**Figure 3B**). Because of their superior biocompatibility, adaptable surface functionalization, and customizable structure and composition, MSNs constitute a significant class of biomedical nanomaterials 68. Upon entering systemic circulation, the platform (T_10_/TPZ@M/IR@L) enabled T_10_ to bind specifically to plasma Tf, forming an *in situ* Tf liposome-PC complex. This approach demonstrated superior targeting efficacy over conventional ligand-modified targeting strategies. Simultaneously, upon exposure to near-infrared irradiation, IR820 activation promoted the infiltration of T_10_/TPZ@M/IR@L into deep breast tumor tissues. Crucially, under near-infrared irradiation, high ROS levels produced by IR820 directly damaged tumor cells and exacerbated tumor hypoxia (**Figure 3C**). Finally, TPZ was activated into cytotoxic metabolites in hypoxic tumor tissues, eliminating 4T1 tumor cells through the synergistic effects of photo-chemotherapy (**Figure 3D**). This strategy could aid the design of multifunctional liposome delivery systems for efficient active targeting and enhanced photo-chemotherapy in breast cancer.

#### 5.1.3. Peptide-modified liposomes enabling photo-immunotherapy synergy

In recent years, combining PDT with immunotherapy has attracted increasing attention as a promising strategy for cancer treatment. Numerous studies have shown that inducing ICD can address the issue of low immune responses in tumor immunotherapy. Compared with drug-induced ICD, PDT offers several advantages, including high selectivity, minimal side effects, precise spatiotemporal control, and the ability to overcome multidrug resistance. More notably, the clinical application of photodynamic therapy (PDT) in oncology has advanced substantially. Several photosensitizing agents approved by the Food and Drug Administration (FDA), including Photofrin and Verteporfin, have been introduced for the treatment of malignancies such as lung and esophageal cancers 69. A cRGD-modified liposomal system including the photosensitiser pheophorbide A (Pa) and the anti-programmed cell death ligand 1 (PD-L1) antibody was created by Qin *et al*. 70. Pa is a naturally occurring second-generation photosensitiser in the chlorophyll derivative family 71. The photosensitizer has a high light-absorption cross-section and is highly phototoxic. These characteristics have made it a good candidate for PDT in various tumours 72. cRGD-modified liposomes enhanced tumour targeting and improved the solubility and biocompatibility of Pa. The cRGD-PaNPs-αPD-L1 group exhibited a more favourable immune microenvironment in 4T1 tumours compared with the other groups. The system raised the activation rate of mature dendritic cells from 0.023% to 0.077% and the infiltration rate of cytotoxic T cells from 0.23% to 0.92%. Therefore, the two were combined in this study to improve the effect of PD-1/PD-L1 inhibition.

#### 5.1.4. Peptide-modified liposomes enabling radio-immunotherapy synergy

**Figure 4:**
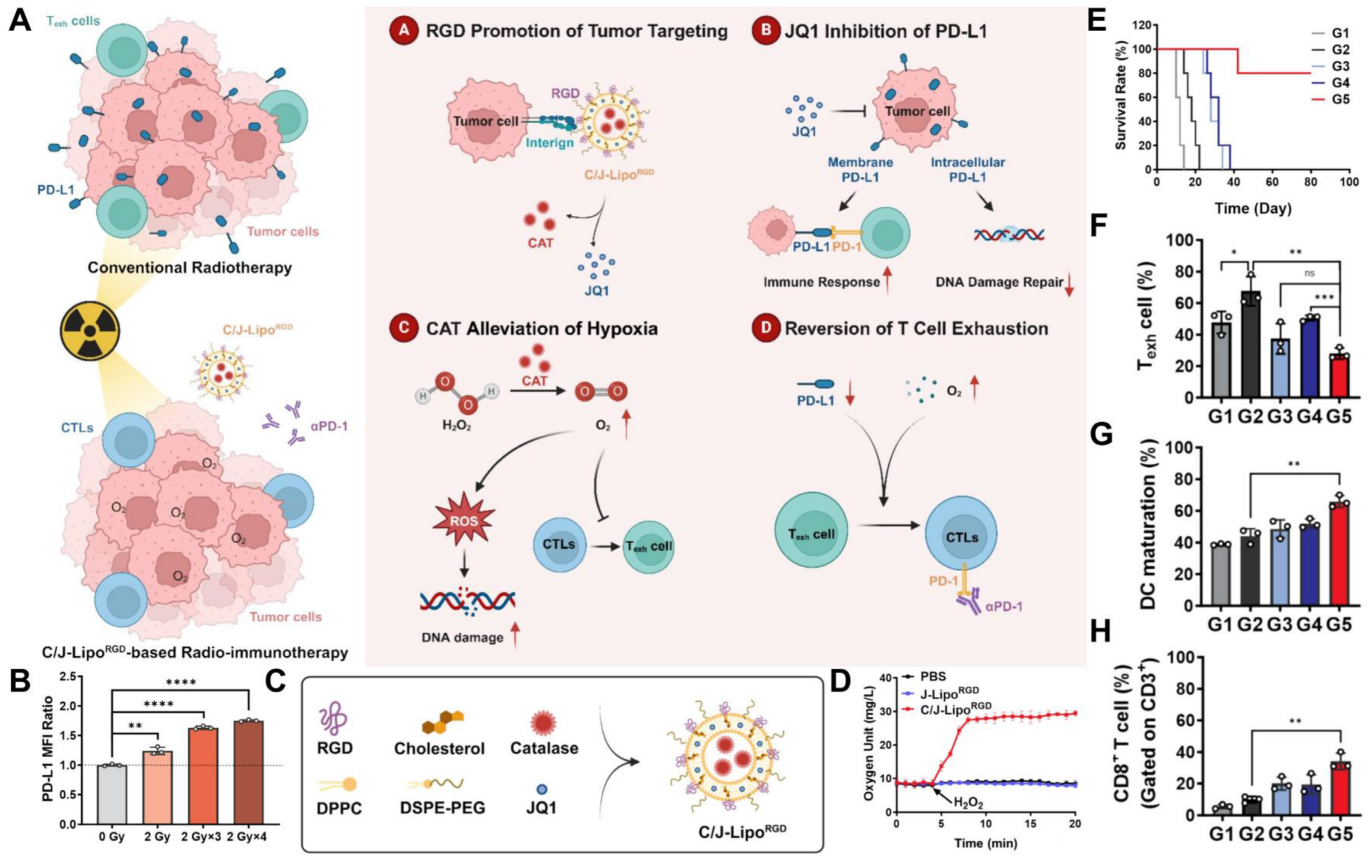
Peptide-functionalized liposomal platforms for cancer radioimmunotherapy. (A) Schematic illustration of C/J-Lipo^RGD^-mediated tumor microenvironment remodeling for potentiating radioimmunotherapy. (B) Flow cytometric analysis of PD-L1 expression in B16-F10 cells 24 h after exposure to different doses of X-ray irradiation. (C) Diagram of cRGD-modified liposomes co-encapsulating JQ1 and catalase for tumor-targeted delivery. (D) Oxygen generation profiles of PBS, J-Lipo^RGD^, and C/J-Lipo^RGD^ solutions following the addition of H₂O₂ at 4 min. (E) Survival curves of B16-F10 tumor-bearing mice subjected to various therapeutic regimens. (F) Flow cytometric detection and quantitative analysis of exhausted T cells, defined by PD-1 expression, within tumor tissues after different treatments. (G) Representative flow cytometry plots and corresponding quantification of mature dendritic cells in tumors following each treatment. (H) Flow cytometric assessment of tumor-infiltrating T cells and the proportion of CD8⁺ T cells among CD3⁺ T cells. Data are presented as mean ± SD (n = 3). *P < 0.05, **P < 0.01, ***P < 0.001. Adapted with permission from 77, Copyright 2025, Elsevier Ltd.

Radiation therapy (RT) is one of the three main kinds of cancer treatment that damages the DNA of cancer cells and stops them from dividing. Although widely used for the treatment of cancer, RT will inevitably induce changes in the TME and cause radiation resistance and immunosuppression. RT is now widely used to enhance the sensitivity and general efficacy of immunotherapy 73-74. Based on the above evidence, RT directly kills malignant cells and, at the same time, remodels the immune environment in tumours by inducing immunogenic cell death, modifying anti-tumour immune responses, and triggering systemic abscopal effects. The efficacy of local RT combined with immunotherapeutic agents, particularly immune checkpoint inhibitors, has been validated in preclinical models and clinical trials 76. Interestingly, Yue *et al*. 77 investigated radiation-induced changes in the TME. The study revealed that X-ray exposure elevated PD-L1 levels, aggravated intratumoral hypoxia, and promoted exhausted T-cell phenotypes, thereby weakening the therapeutic benefit of fractionated radiotherapy (**Figure 4B**). In response to these radiation-associated limitations, to accomplish tumor-selective accumulation and control the tumor microenvironment, the scientists created C/J-Lipo^RGD^, a cRGD-functionalized liposomal nanoplatform co-loaded with catalase and the JQ1. (**Figure 4A, C**). Catalase converted excessive tumor H₂O₂ into oxygen and water, thereby alleviating hypoxia and improving local oxygen availability (**Figure 4D**). After cellular uptake, JQ1 suppressed PD-L1 expression, which inhibited PD-1/PD-L1-mediated immunosuppression and interfered with DNA damage repair. Flow cytometric profiling indicated that radiotherapy alone modestly increased lymph node dendritic cell maturation to 44.13%, whereas the triple regimen consisting of C/J-Lipo^RGD^ and αPD-1 markedly elevated this proportion to 70.10%. In parallel, treatment with C/J-Lipo^RGD^ increased intratumoral CD3⁺CD8⁺ T-cell infiltration and enhanced the expression of TNF-α and IFN-γ. Overall, C/J-Lipo^RGD^-based radioimmunotherapy reshaped the TME by relieving hypoxia, suppressing PD-L1 signaling, reversing T-cell exhaustion, promoting DCs maturation, and strengthening CD8⁺ T-cell-mediated antitumor immunity (**Figure 4E-H**). These coordinated effects ultimately improved the therapeutic efficacy of radiotherapy combined with immune checkpoint blockade.

In summary, peptide-modified liposomal drug delivery systems enable targeted cancer therapy by enhancing intracellular uptake, improving tumor penetration, and reducing off-target effects. The integration of peptides with liposomes has shown promise in chemo-immunotherapy, photo-chemotherapy, and other combination therapies. However, challenges remain, including optimizing peptide stability and overcoming immune suppression within the TME.

### 5.2. Glycosylation

Tumor-associated alterations in cell-surface glycosylation are commonly manifested as the generation of truncated glycan structures, increased expression of highly branched N- and O-linked glycans, and dysregulated fucosylation or sialylation profiles 78. Because malignant cells often have enhanced glucose uptake and abnormal expression of glucose transporters and sodium-dependent glucose cotransporters, these carbohydrate-related pathways have become attractive targets for tumor-selective therapy 79. Glycosylation is a general kind of post-translational modification that adds carbohydrate chains to proteins and lipids to change their shape, stability and other biological functions. Changes occur in many areas during tumour development that alter the glycan pattern of cancer cells compared with those in normal tissues 80. For example, tumour cells may have an increased amount of certain carbohydrate groups, such as Gal-β-D-mannose and N-acetyl-D-glucosamine, and a reduced quantity of other glycan structures 81, 82. Based on the above, glycan-containing ligands have been gradually incorporated into drug delivery systems for glycosylation-directed targeting. Another therapeutic strategy is to inhibit glycosyltransferases or the glycosylation pathway in tumours that have been aberrantly activated. In particular, inhibition of O-GlcNAc transferase has been reported to suppress tumor growth and metastatic progression in several malignancies 83, 84. Moreover, differences in the composition, density, and spatial distribution of glycans between malignant and healthy cells provide a molecular foundation for cancer-specific recognition and intervention. Therefore, exploiting tumor-associated glycosylation abnormalities represents a promising route for active targeting, and continued research in this area may support the development of more precise anticancer therapies 85, 86. Liposomes are especially suitable for this purpose because their surfaces can be readily modified with defined carbohydrate ligands. By identifying tumor-associated biomarkers, these glycan-functionalized liposomal systems enhance targeted delivery and may find use in cancer imaging and diagnostics.

#### 5.2.1. Glycosylated liposomes enabling photo-immunotherapy synergy

Mannose (Man) is a naturally occurring monosaccharide found in various plant sources, including citrus peels, peaches, and apples, as well as in biological materials such as ivory palm kernels and yeast 87. Mannosyl groups are commonly used to modify anticancer drugs, such as platinum-based drugs, as well as drug carriers; proteins and chitosan are typical examples, and they show good potential in drug delivery systems. Maillard reaction provides a feasible strategy for preparing drug delivery platforms by coupling mannose with amino-group-containing materials, such as liposomes, chitosan and protein-based carriers 88. Liposomes modified with mannose derivatives actively target hepatocellular carcinoma cells by binding to mannose receptors (MR) and have shown good results in targeted delivery 89. Four members make up the extremely effective endogenous receptor system known as the mannose receptor family: MR (CD206), phospholipase A2 receptor (PLA2R), Endo180 (CD280), and DEC-205 (CD205) 90. Its functions include clearing endogenous molecules, promoting antigen presentation, and regulating cell activation and trafficking 91, 92. Additionally, MR is highly expressed in splenic and alveolar macrophages 93. Accordingly, mannose-functionalized drug delivery platforms have attracted considerable interest as promising therapeutic carriers, owing to their favorable delivery performance, enhanced targeting potential, and relatively low toxicity. A recent landmark study demonstrated that trimannose conjugation markedly improved the pulmonary macrophage delivery of inhaled oligonucleotides, establishing this platform as the first mannosylated therapeutic candidate developed for COVID-19 94.

Recent advances in cancer immunotherapy have increased interest in tumor vaccines. Tumor vaccines are characterized by durable immune memory and antigen-specific immune responses, and they have demonstrated promising therapeutic efficacy in clinical trials 95. Among these, *in situ* vaccination generates endogenous antigens for autologous tumor cells *in vivo*, eliminating the need to identify and isolate tumor-associated antigens (TAAs), thereby eliciting a broad immune response 96, 97. In PTT, near-infrared (NIR) light, with its strong tissue penetration, kills tumor cells and releases TAAs. As a result, localized PTT combined with immune activation from *in situ* vaccines achieves superior anticancer effects. Based on photothermal-immunotherapy, Li *et al*. 98 proposed a photothermal-triggered *in situ* vaccine consisting of acid-responsive liposome-coated polydopamine (PDA) nanoparticles, modified with Man and loaded with resiquimod (R848). First, acid-responsive liposomes (PMRL) were cleaved in the acidic TME at the tumor site, exposing PDA-Man@R848 nanoparticles. These nanoparticles not only mediated photothermal conversion but also induced ICD and promoted the release of TAAs. Meanwhile, the surface of PDA-Man@R848 nanoparticles was modified with Man. Relative to the PBS-treated cohort, administration of PMRL markedly enhanced T-cell accumulation within tumors, increasing CD4⁺ and CD8⁺ T-cell infiltration by 2.01- and 2.15-fold, respectively. Furthermore, ELISA analysis showed that the PMRL group had lower serum TGF-β levels and higher IFN-γ and TNF-α secretion than the other groups, indicating that PMRL induced a robust pro-inflammatory immune response. Man-modified PDA@R848 nanoparticles promoted the maturation of DCs, enhanced antigen cross-presentation, and strengthened anticancer adaptive immunity. In addition, the vaccine effectively inhibited distant tumor recurrence and lung metastasis in the 4T1 model by inducing long-term immune memory.

#### 5.2.2. Glycosylated liposomes enabling chemo-immunotherapy synergy

Glucose serves as an essential cellular energy substrate, and its uptake is primarily mediated by glucose transporters (GLUTs), which are broadly expressed on the surface of most cell types. This results in the overexpression of GLUT-1 on tumor cell surfaces, sustaining their high glucose uptake. Consequently, GLUT-1 is considered a potential target for anticancer therapy. GLUT-1-specific carbohydrates, such as glucose, 2-deoxyglucose, and glucosamine analogues, can be conjugated to liposome drug delivery vehicles for transport via GLUT-1 99. Fu *et al*. 100 co-encapsulated 20(S)-protopanaxadiol (PPD) and cannabidiol (CBD) within n-dodecyl β-D-maltoside (Mal)-modified liposomes to evaluate their synergistic anticancer effect on breast cancer. The Mal surface contains two glucosyl residues, which mediate tumor-targeting functionality. Protopanaxadiol (PPD), a bioactive constituent originating from the traditional Chinese medicinal herb *ginseng*, exhibits diverse pharmacological activities and considerable therapeutic potential 101. Cannabidiol (CBD), one of the major phytochemicals isolated from *Cannabis sativa*, has also been extensively investigated for applications in neurodegenerative disorders 102. Notably, neither PPD nor CBD alone displays strong antitumor activity. However, when these two agents were co-loaded into maleimide-modified liposomes, the resulting combinational formulation produced a pronounced therapeutic effect, achieving an 82.2% tumor suppression rate.

#### 5.2.3. Glycosylated liposomes enabling ferroptosis–immunotherapy synergy

Ferroptosis is a relatively new mode of cell death. Stockwell's laboratory in 2012 discovered a new kind of regulated cell death that occurs when iron-dependent accumulation of lethal membrane-localized lipid peroxides takes place 103, 104. Recently, modulators of ferroptosis have been used to inhibit the development and spread of cancer, and thus have attracted much attention in etiology and therapy research 105, 106. Therefore, ferroptosis is now regarded as a good target for cancer therapy, and in combination with other drugs, its effect can be enhanced to address problems such as the reduced efficacy of traditional treatments, drug resistance in tumours, and recurrence. Ferroptosis-immunotherapy can suppress cancer cells directly and boost the immune response via ICD to build a strong anti-cancer immunity 107. Ferroptosis in the tumour microenvironment is regulated by complex cross-talk among malignant cells and immune cells 108. TAAs and DAMPs are released by ferroptotic cells to promote DC maturation and T cell-mediated anti-tumor immunity. MHC-I recognition activates the JAK1/STAT1 pathway in tumour cells by CD8^+^ T cells, inhibits System Xc function, decreases GSH and GPX4, and thus increases susceptibility to ferroptosis 109, 110. Furthermore, the ferroptosis pathway in immunosuppressive cell populations can be inhibited to reduce their tumour-promoting effects and alleviate immune suppression 111. Not only do some ferroptosis-inducing agents cause ferroptotic death in M2-like macrophages, but they also promote their phenotypic switching to antitumor M1 macrophages. These M1-polarised macrophages can produce hydrogen peroxide and thus enhance Fenton chemistry in tumours, forming a positive feedback loop that further promotes ferroptosis 112. Combine ferroptosis and immunotherapy to offer a new way of extended tumour suppression and improved treatment effect. Functionalized Drug Delivery Systems offer multiple advantages for this cooperative strategy. Glycosylated functionalized liposomes for tumour targeting through ligand-receptor interactions. At the same time, TME-responsive systems can achieve space-time-controlled drug release by using acidic pH, high GSH levels or enzyme overexpression 113.

For example, Gao *et al*. 114 reported a fucose-modified liposome that establishes a positive loop between ferroptotic therapy and immunotherapy. Fucose, a hexose sugar also known as 6-deoxy-L-galactose, is widely distributed in glycoproteins and glycolipids of living organisms. Liposomes are surface-modified with fucose, which binds to overexpressed mannose receptor C-type 1 (MRC1 or CD206) on tumor cell surfaces, mediating active targeting 115. Interestingly, researchers engineered a lipid (DAPC) with phosphatidylcholine as its polar head and two arachidonic acid moieties as hydrophobic tails. This lipid responded to high ROS levels in the TME, inducing ferroptosis-specific peroxidation of DAPC and triggering the release of CpG ODNs. CpG ODN, as an immunostimulant, further promoted DC maturation and enhanced the effector function of CD8^+^ T cells. Additionally, IFN-γ released by activated CD8^+^ T cells promoted ferroptosis in cancer cells by inhibiting SLC7A11 and GSH biosynthesis. In the 4T1 tumor-bearing mouse model, this liposome (DAPC-Fuc/CpG) effectively suppressed tumor growth and demonstrated optimal therapeutic efficacy across all treatment groups. Overall, the DAPC-Fuc/CpG triple-action platform, integrating active targeting, ROS-responsive release, and selective lipid peroxidation, offered a promising strategy for coordinating ferroptosis-immunotherapy.

In summary, advances in glycobiology, glycan engineering, and glycomics have substantially accelerated the biomedical application of carbohydrate-based strategies. These sugar structures enable efficient active targeting of various tumor cells and can be used to deliver different types of drugs, genes, and therapeutic RNA molecules. Glycan-modified liposome delivery systems emphasize selective targeting of cancer cells via specific interactions with overexpressed glycan receptors.

### 5.3. Aptamer

In the early 1990s, two independent research groups made the co-discovery of aptamer technology and ushered in a new era of molecular recognition 116. These synthetic oligonucleotides are called aptamers, and they are obtained by the technique of systematic evolution of ligands by exponential enrichment 117. Aptamers are relatively small, functional single-stranded DNA or RNA oligonucleotides that can fold into specific three-dimensional shapes and bind to their targets with high affinity and specificity. Aptamers are taken up by tumour cells via clathrin-mediated endocytosis and micropinocytosis 118, 119. Aptamers have a wide range of targets and can bind to ions, small molecules, peptides, proteins, cells and tissues. Both DNA and RNA aptamers have strong binding affinity for multiple targets, as shown in 120, 121. Aptamers serve as specific ligands in the construction of target-delivery systems for anticancer nanomedicine frequently.

#### 5.3.1. Aptamer-modified liposomes enabling photo-immunotherapy synergy

AS1411 aptamers are hydrophilic, guanosine-rich single-stranded DNA molecules. AS1411 has a length of 26 nucleotides and is a specific G-quadruplex. AS1411 has an interesting specific nucleotide sequence: 5'-GGTGGTGGTGGTGGT-3'. This peptide sequence can selectively recognise and bind to receptors expressed on the surface of cancer cells, thus having strong tumour-targeting ability 122. AS1411 is a typical example of a receptor that binds to tumours, such as ribosomal protein S6, epithelial cell adhesion molecules, and nucleolin (a nucleic acid transporter). AS1411 aptamers have excellent specificity, can enhance receptor-mediated cellular uptake and are less prone to off-target toxicity. Significantly, the G-quadruplex structure of the AS1411 aptamer binds to DNA or RNA in cancer cells and thus inhibits their transcription and replication. Thus, inhibiting the growth of cancer cells and inducing apoptosis 123. AS1411 can modify the function of T cells, natural killer (NK) cells and dendritic cells, etc., as shown in some studies. AS1411 can be combined with immune checkpoint inhibitors to boost the tumour-killing effect of the immune system and inhibit immune evasion 124. AS1411 can be used in conjunction with a liposomal delivery system to fully realise its strong effects, and through multimodal therapy, it is now expected to serve as a high-precision and adaptable platform for breast cancer treatment.

Based on AS1411 aptamer modification, Li *et al*. 125 constructed a functionalized liposome loaded with Golgi-targeted carbon dots (CDs)-A-chain (RTA) conjugates and the photosensitizer pheophorbide a (PPa), enabling targeted synergistic chemotherapy and PDT. The liposome (AS1411/lip/PPa/C-R) preferentially accumulated at tumor sites due to the high affinity between nucleolin and the AS1411 aptamer. It was then internalized and transported to lysosomes. Under near-infrared laser irradiation, PPa generated large amounts of ROS for PDT. Concurrently, the CDs-RTA conjugate escaped from lysosomes and moved to the Golgi apparatus, where RTA dissociated from CDs. RTA was then transported retrogradely to the endoplasmic reticulum and ribosomes, where it irreversibly inhibited protein synthesis and induced cell death, thereby exerting potent chemotherapeutic effects. In conclusion, the bimodal synergistic targeted liposomal therapy system developed in this study achieved highly effective photodynamic-chemotherapy for breast cancer.

**Figure 5:**
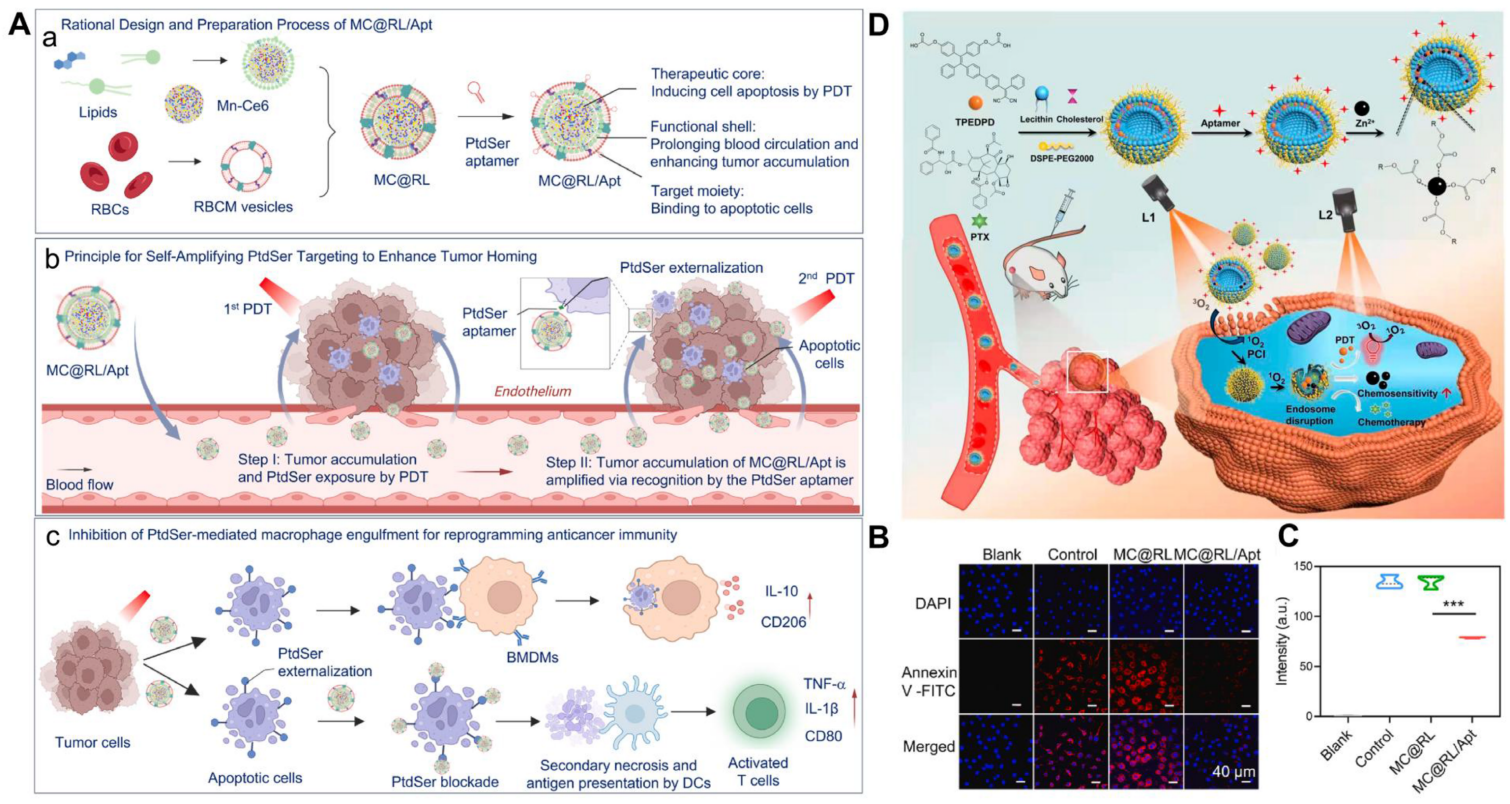
Aptamer-modified liposomal drug delivery systems for combination cancer therapy. (A) Diagrammatic representation of a self-amplifying PtdSer-targeted approach intended to improve tumor treatment and modify antitumor immunity by blocking efferocytosis and tumor homing. (a) An example of MC@RL/Apt' s logical creation and preparation. (b) When exposed to a 660 nm laser, MC@RL/Apt causes tumor cell death and encourages PtdSer externalization on the tumor cell membrane. This draws more MC@RL/Apt to the tumor location and creates a self-reinforcing accumulation effect. (c) By inhibiting macrophages from eliminating apoptotic cells and encouraging macrophage repolarization toward a pro-inflammatory M1 phenotype, MC@RL/Apt triggers potent anticancer immune responses. (B, C) Quantitative study of Annexin V-FITC staining in apoptotic CT26 cells after various treatments, along with representative confocal fluorescence pictures. The blank control was CT26 cells that had not been treated. Adapted with permission from 133, Copyright 2026, Elsevier Ltd. (D) Schematic representation of PTX/PS-Zn@Lip-Apt preparation and light-triggered therapeutic activation. The first irradiation phase (L1) was used to improve cellular internalisation by photochemical internalisation (PCI) after the accumulation of PTX/PS-Zn@Lip-Apt nanoparticles inside tumor tissues. Subsequently, a second light exposure (L2) was introduced to promote robust reactive oxygen species (ROS) generation for improved therapeutic efficacy. Adapted with permission from 126, Copyright 2025, Elsevier Ltd.

Similarly, Gao *et al*. 126 developed a prostate cancer-targeted liposomal system (PTX/PS-Zn@Lip-Apt) by co-loading PTX and Zn²⁺ and using aggregation-induced emission (AIE) to facilitate coordination between Zn²⁺ and AIE-associated carboxyl groups (**Figure 5D**). Notably, the investigators adopted a photochemical internalization (PCI) approach to achieve light-triggered, spatially controlled release of therapeutic cargos, including drugs and genetic materials. Studies showed that introducing the AS1411 aptamer mediated selective uptake by PC3 prostate cancer cells, while PCI enhanced endosomal escape efficiency. These findings were validated by tumor imaging, providing strong evidence for the efficacy of photodynamic-chemotherapy mediated by PTX/PS-Zn@Lip-Apt.

#### 5.3.2. Aptamer-modified liposomes enabling chemo-immunotherapy synergy

Phosphatidylserine (PtdSer) is a negatively charged phospholipid consisting of a serine head group linked to a glycerophospholipid backbone. Under normal conditions, it resides primarily on the inner surface of cell membranes. In tumor cells, particularly in advanced cancer cells, PtdSer is frequently exposed on the outer leaflet of the cell membrane. The outward exposure of PtdSer on tumor cell membranes is a hallmark of tumor cell transformation and is closely linked to the invasive and metastatic properties of tumor cells 127-129. Additionally, PtdSer is externalized to the outer leaflet of the plasma membrane during apoptosis, where it functions as an “eat-me” signal that promotes the recognition and engulfment of dying cells by macrophages and other professional phagocytes. Nevertheless, macrophages' efferocytosis of apoptotic cells might start immunosuppressive signalling, favoring their polarisation toward an anti-inflammatory M2-like phenotype and encouraging the production of TGF-β and interleukin-10 (IL-10) 130-132. Together, these processes limit effective antitumor immune activation and help create an immunosuppressive tumor microenvironment.

Based on this, Ren *et al*. 133 developed a PtdSer aptamer-functionalized, red blood cell membrane-camouflaged Mn-Ce6 nanocomplex, termed MC@RL/Apt, to facilitate tumor homing and suppress efferocytosis, thereby reshaping antitumor immunity (**Figure 5A**). By utilizing erythrocyte membrane camouflage, this platform achieved extended systemic circulation. Upon 660 nm laser irradiation, Mn-Ce6 induced apoptotic death in tumor cells and enhanced PtdSer externalization, which further promoted progressive “snowball-like” tumor accumulation through specific recognition by PtdSer aptamers (**Figure 5B, C**). Simultaneously, PtdSer blocked the interaction between PtdSer and the Tim-4 receptor on the macrophage surface, thereby inhibiting phagocytosis. This increased M1-type marker expression (iNOS, TNF-α) threefold while decreasing M2-type markers (Arg-1, IL-10) by 60%. The process reprogrammed macrophages into the pro-inflammatory M1 type and activated antigen presentation in dendritic cells. The “killing two birds with one stone” strategy resulted in MC@RL/Apt accumulation at the tumor site being 1.46 times that of the non-functionalized group. *In vivo* experiments showed that these functionalized liposomes reduced tumor volume by 72% in the CT26 tumor model, increased CD8^+^ T cell infiltration fourfold, and induced a systemic anticancer immune response. This study pioneered the integration of PtdSer’s “targeting” and “immunomodulatory” functions, overcoming the limitations of traditional nanomedicines’ “passive accumulation.” The self-amplifying design addressed tumor heterogeneity challenges, while remodeling the immune microenvironment laid the foundation for combining chemotherapy with immunotherapy.

In conclusion, aptamers offer several advantages: simple synthesis, structural versatility, high chemical stability, strong tissue penetration, and low immunogenicity. In preclinical studies and recent clinical applications, aptamers have shown promise as ligands for targeted treatment of tumors.

### 5.4. Other ligand modifications

#### 5.4.1. Folic acid

Folic acid is a water-soluble vitamin found in foods like leafy green vegetables, legumes, nuts, and certain animal livers 134. It has significant potential as a targeting ligand in drug delivery systems due to its high binding affinity for folate receptors (FRs) 135, 136. FRs are cysteine-rich cell surface glycoproteins present in three isoforms: FR-α, FR-β, and FR-γ. Among these, FR-α is the most studied isoform, showing low expression in normal cells but being overexpressed in malignancies like lung, colon, breast, and ovarian cancers 137. Research shows that FR density increases with cancer stage and grade 138-140. Therefore, FR-α can serve as a target for designing active cancer-targeting therapies. For instance, folate can be covalently attached to liposomes.

Extensive research shows that enhanced immunotherapy efficacy requires sufficient infiltration by NK cells and CD8^+^ T cells. Interleukin-15 (IL-15), a stimulatory cytokine, enhances the proliferation and activation of natural killer (NK) and CD8^+^ T cells by efficiently binding to the IL-15 receptor 141. Liu *et al*. 142 designed FA-PEG-modified liposomes for the separate delivery of plasmid IL-15 (pIL-15) and gemcitabine (GEM) (FPCL@pIL-15 + FPGL). Both FPCL@pIL-15 and FPGL exhibited symmetrical spherical structures with particle sizes of 150 nm and 120 nm, respectively, demonstrating ideal penetration and accumulation in tumor tissues. After tumor cell-specific uptake of FPCL@pIL-15, the encapsulated pIL-15 escaped from endosomal vesicles into the cytoplasm, facilitating transfection and expression and thereby significantly promoting the proliferation and activation of NK cells and T cells. Concurrently, FPGL upregulated NKG2DLs, mediating the NKG2D/NKG2DL axis to enhance NK cell recognition and reduce immune escape. It also promoted CD8^+^ T cell activation via the ICD effect. Activated NK and CD8^+^ T cells delivered potent cytotoxic effects against cancer cells by increasing granzyme B, IFN-γ, and TNF-α expression. Crucially, FPCL@pIL-15 and FPGL-mediated chemo-immunotherapy demonstrated promising antitumor efficacy in a mouse 4T1 tumor model.

Light-responsive liposomal platforms have been engineered to enable spatiotemporally controlled release of drugs in response to external light stimulation. Chitgupi and colleagues 143 developed FA-modified liposomes co-loaded with light-sensitive porphyrin-phospholipid (PoP) and doxorubicin (Dox) for ovarian cancer therapy. *In vitro* co-incubation experiments showed that FA-conjugated liposomes had superior uptake rates compared to non-FA-modified liposomes when incubated with human KB cancer cells overexpressing FRs. Interestingly, Dox accumulation under phototherapy was 4-6 times higher than that achieved with chemotherapy alone. Furthermore, *in vivo* xenograft mouse experiments showed delayed tumor growth and improved survival rates in the functionalized liposome-treated group compared to other groups.

In short, folate-conjugated liposomes are an effective strategy for combination cancer therapy. These modified liposomes selectively target tumor cells, enhance intracellular delivery, and improve stability, biocompatibility, the ability to overcome drug resistance, and pharmacokinetic properties.

#### 5.4.2. Transferrin

TfR is upregulated on tumor cell surfaces as a result of the unchecked growth and multiplication of cancer cells, which need much more iron than normal cells, according to several studies. TfR is widely expressed in most cell types and is involved in iron uptake. However, various tumor cells and brain capillary endothelial cells overexpress TfR at levels at least 100 times higher than in normal cells 144, 145. Therefore, TfR is an attractive target for developing cancer therapy ligands. Researchers have explored using Tf as the natural ligand for TfR, enabling drug delivery to target cells via Tf-TfR receptor-mediated endocytosis. This approach represents an emerging strategy for targeted cancer therapy 146. Tf is a glycoprotein primarily found in the bloodstream, with the main function of transporting iron ions (Fe³⁺) to cells throughout the body. Compared to traditional antibodies or other ligands, as a naturally occurring biomolecule, Tf is less likely to elicit an immune response and has minimal immunogenicity. This makes Tf-modified drug delivery systems safer for practical use, reducing the risk of immune rejection. In addition, transferrin-modified drug delivery systems offer other advantages, including reduced systemic drug distribution, higher drug concentrations at the target site, and enhanced synergistic toxicity against tumor cells 147, 148.

**Figure 6:**
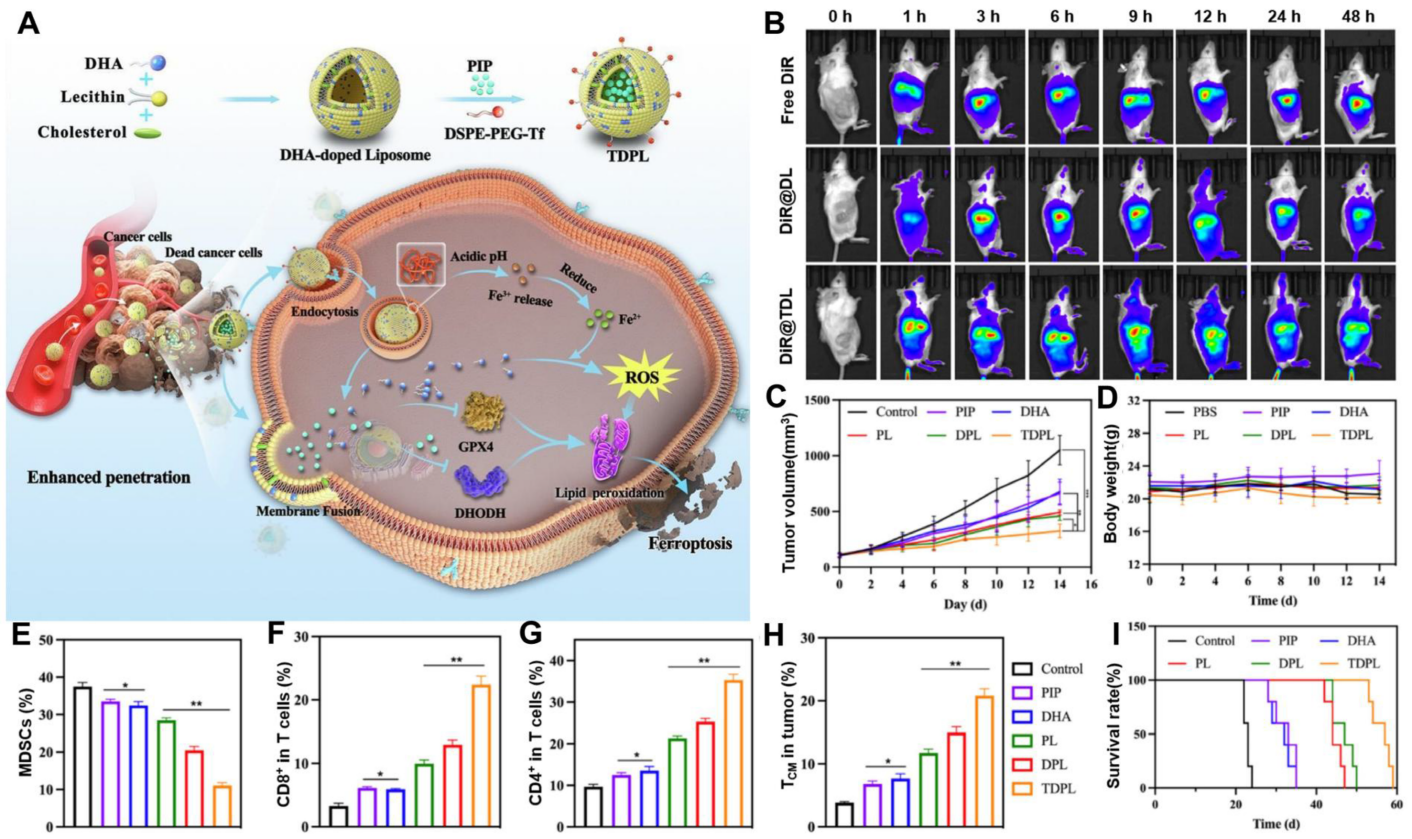
Transferrin-modified liposomal drug delivery systems for cancer ferroptosis-immunotherapy. (A) Schematic diagram of the preparation of transferrin-modified liposomes (TDPL) and therapeutic mechanism. (B) *In vivo* biodistribution of free DiR, DHA-containing DiR-loaded liposomes (DiR@DL), and transferrin-functionalized DHA-containing DiR-loaded liposomes (DiR@TDL) in 4T1 tumor-bearing mice following intravenous administration. (C) Mean tumor volume changes in mice receiving different treatments, including control, PIP, DHA, PL, DPL, and TDPL groups (n = 5). (D) Body weight variations of mice throughout the treatment period (n = 5). The proportions of (E) CD8⁺ T cells among total T cells, (F) CD4⁺ T cells among total T cells, (G) myeloid-derived suppressor cells (MDSCs), and (H) central memory T cells (T_CM_) (n = 3) indicate the *in vivo* immune regulation after different therapeutic treatments. (I) Kaplan-Meier survival analysis of five mice given various treatments. *P < 0.05, **P < 0.01, ***P < 0.001. Adapted with permission from 149, Copyright 2025, Elsevier Ltd.

Huang *et al*. 149 developed Tf-modified liposomes (TDPL) doped with the unsaturated fatty acid docosahexaenoic acid (DHA) as a lipid peroxidation inducer and loaded with piperine (PIP) to enable effective ferroptosis-chemo-immunotherapy (**Figure 6A**). In this platform, Tf served a dual role, acting as both a ligand targeting TfR and a Fe^3+^ ionophore. Triggered by the low pH in the lysosome, Tf-bound Fe^3+^ was released and reduced to Fe^2+^, which subsequently catalyzed the oxidation of unsaturated lipids in addition to participating in the Fenton reaction (**Figure 6B**). Meanwhile, DHA incorporated into the lipid bilayer fuses with the cell membrane, inactivating GPX4 and inducing lipid peroxidation. In addition, piperine (PIP), an alkaloid found primarily in *Piper longum*, exerts potent anticancer effects by inducing cell cycle arrest and apoptosis, suppressing signaling protein expression, and targeting multiple cancer-related signaling pathways. *In vitro* experiments showed that this liposome reduced GPX4 and DHODH levels, effectively overcoming the GPX4-mediated ferroptosis defense pathway. Concurrently, piperine (PIP) downregulated DHODH expression, thereby further promoting lipid peroxidation. *In vivo* studies using 4T1 tumor-bearing mice demonstrated that the TDPL-treated group achieved the most pronounced antitumor efficacy and the longest survival duration among all experimental groups (**Figure 6C, D, I**). Notably, analysis of tumor-draining lymph nodes revealed that TDPL markedly increased dendritic cell maturation to 42.7%, which was substantially higher than that observed in the other treatment groups. These findings suggested that TDPL effectively induced immunogenic cell death and promoted dendritic cell activation *in vivo* (**Figure 6E-H**). Overall, this work provides a useful reference for integrating polyunsaturated fatty acids with natural bioactive compounds in multimodal cancer therapy.

#### 5.4.3. Nanobody

In the design of functionalized liposomes, nanobodies have emerged as a novel class of ligand molecule. They are derived from the variable domains of naturally occurring heavy-chain-only antibodies in camelids and fish 150-153. Specifically, nanobodies are particularly useful as they are roughly 10 times smaller (12-15 kDa) than conventional full-length antibodies, contributing to better tumor-penetrating characteristics 154. Nanobodies are highly stable under acidic pH conditions and at high temperatures, allowing them to withstand the harsh tumor microenvironment. These properties make them particularly suitable for modification, including radiolabeling and chemical conjugation to fluorescent dyes, liposomes, photosensitizers, and immunomodulatory molecules 155. More importantly, the simple structure and easy production of nanobodies enable diverse molecular engineering strategies. Consequently, nanobodies can be engineered in multivalent forms to enhance stability and affinity, or fused with Fc domains to confer effector functions.

Bouma and colleagues 156 developed a novel vaccine formulation by conjugating CD169- or DC-SIGN-specific nanobodies to liposomes. This design enabled high-affinity targeting and promoted selective uptake by antigen-presenting cells (APCs). *In vivo* and *in vitro* studies showed that both nanobody-conjugated liposomes exhibited increased uptake compared to control liposomes or those bearing the native ligands for CD169 and DC-SIGN. This enhanced uptake increased T cell activation by human APCs and stimulated naive T cell activation in mouse models.** Table 2** summarizes common ligands for functionalized liposome modification, corresponding receptors, and typical target cells.

### 5.5. Multiple ligand modifications

Single-target-modified liposomes have reached a relatively mature stage of development, but their further application faces limitations. Single-target modifications targeting cancer cell receptors often cause non-selective toxicity, as normal cells also express these receptors. To address this issue, researchers have explored multi-target modification strategies for liposomes to improve the targeting precision of liposomal drug delivery systems.

#### 5.5.1. Glycosylation-Peptide

**Figure 7:**
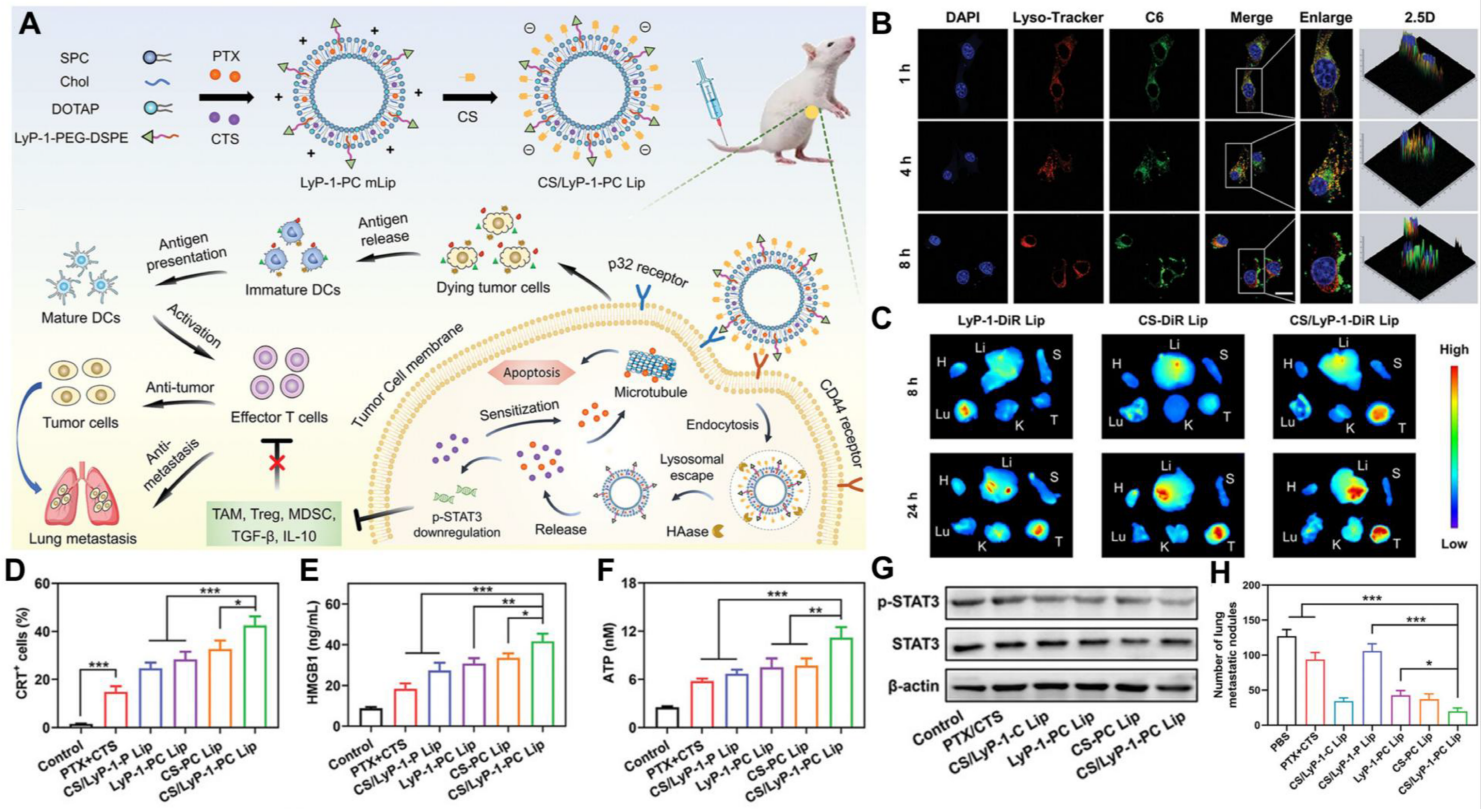
Peptide/glycans dual-modified liposomal drug delivery systems for cancer chemo-immunotherapy. (A) Schematic illustration of the preparation of the dual-modified LyP-1/chondroitin sulfate liposomal system (CS/LyP-1-PC Lip), together with its intracellular trafficking behavior and proposed therapeutic mechanism. The CS/LyP-1-PC Lip formulation enhances chemoimmunotherapy against triple-negative breast cancer (TNBC) by inducing tumor immunogenicity and suppressing STAT3 signaling. (B) Confocal laser scanning microscopy (CLSM) images showing the lysosomal escape behavior of CS/LyP-1-C6 Lip in 4T1 cells at 1, 4, and 8 h. (C) *Ex vivo* fluorescence images of major organs, including the heart (H), liver (Li), spleen (S), lung (Lu), and kidney (K), as well as tumor tissue (T), at 8 and 24 h after injection. (D) Quantitative evaluation of the expression of calreticulin (CRT) in 4T1 cells. (E) HMGB1 release and (F) ATP secretion after targeted liposomal formulation treatment (n = 3). (G) Western blot examination, STAT3 and phosphorylated STAT3 (p-STAT3) expression. (H) Antimetastatic activity in a metastatic tumor model, as assessed by the total number of pulmonary metastatic nodules after different treatments (n = 5). *P < 0.05, **P < 0.01, ***P < 0.001. Adapted with permission from 25, Copyright 2023, Wiley-VCH GmbH.

Dual-targeted liposomal drug delivery systems based on glycosylation and peptide co-modification for cancer chemo-immunotherapy combinations have recently gained attention in research. LyP-1 (CGNKRTR) is a peptide that binds to PI3K-receptor complexes on the surface of cancer cells and has high selectivity and affinity. A peptide can help a drug enter the tumour cells by binding to receptors on the surface of the tumour cells and thus increase the therapeutic effect of the drug. LyP-1 is expected to target cancer cells and promote the delivery of drugs to the tumour site 157. Chondroitin sulfate (CS) is an anionic polysaccharide in the sulfated glycosaminoglycan family derived from animal cartilage. CS has the above anti-cancer, antioxidant, anti-inflammatory and immunomodulatory properties and can be used to treat various diseases 158, 159. CS also has the attributes of biocompatibility, degradability, mucosal adhesion and hydrophilicity, and has been widely used in biomedicine 160. CS has carboxyl, hydroxyl and amino functional groups that can be modified with hydrophobic components, therapeutic agents or probes to develop various drug delivery systems flexibly 161. CS has a high affinity for the CD44 receptor and is thus taken up by the cell via receptor-mediated endocytosis. Cluster of differentiation 44 (CD44) is a cell surface glycoprotein overexpressed in various solid tumors, including pancreatic, breast, ovarian, brain, and lung cancers 162. CD44 is primarily involved in cell proliferation, cell-to-cell interaction, cellular migration, and immune response generation. Thus, CS serves as an effective ligand targeting the CD44 receptor. Luo *et al*. 25 designed a dual-ligand-modified liposomal system functionalized with the LyP-1 peptide and CS and co-loaded with PTX and the STAT3 inhibitor cryptotanshinone to achieve combined chemotherapy and immunotherapy for triple-negative breast cancer (TNBC) (**Figure 7A**). After intravenous injection, the liposome accumulated in tumor tissue through p32/CD44 dual-receptor-mediated active targeting and was subsequently internalized by 4T1 tumor cells (**Figure 7B, C**). As shown in **Figure 7D-F**, treatment of 4T1 cells with CS/LyP-1-P increased CRT expression, HMGB1 release, and ATP secretion. These results showed that CS/LyP-1-P effectively activated antitumor immunity *in vivo* by inducing ICD by PTX-based chemotherapy. Concurrently, cryptotanshinone downregulated STAT3 expression, reduced the secretion of immunosuppressive factors, decreased the accumulation of immunosuppressive cells, and reversed the immunosuppressive tumor microenvironment (**Figure 7G**). Importantly, in the 4T1 tumor-bearing mouse model, CS/LyP-1-PC Lip exhibited significant antitumor effects and effectively inhibited lung metastasis (**Figure 7H**). This work established a multifunctional liposomal drug delivery platform that modulated the TME, offering a potential strategy for chemo-immunotherapy in TNBC. Similarly, Yuba and colleagues 163 incorporated pH-responsive polysaccharide derivatives and a glycopeptide containing multiple mannose residues (Man9GlcNAc2-Asn) into liposomes to balance cell uptake via lectin-mediated mechanisms with antigen delivery capabilities in the cytoplasm. Man9GlcNAc2-Asn served as a ligand for antigen-presenting cell lectins (e.g., DC-SIGN and DC-SIGNR), significantly enhancing DC uptake of liposomes and improving cross-presentation efficiency. Compared to unmodified liposomes, co-modified liposomes bearing both glycopeptide and pH-responsive polysaccharide moieties cooperatively promoted cellular association via lectin binding and scavenger receptor recognition. Concordant *in vitro* and *in vivo* results confirmed the strong adjuvant activity of this system, as reflected by increased populations of splenic dendritic cells, M1-polarized macrophages, and effector T cells. In addition, Haas and colleagues 164 investigated the immunostimulatory effects of liposomal vaccines modified with the sugar chain Le^Y^ and synthetic SLP peptides. Results showed that Le^Y^ modification enhanced the targeting and uptake of liposomes by DCs, while Le^Y^-modified liposomes exhibited potent antitumor effects in the B16-OVA tumor model. Furthermore, binding of synthetic SLP peptides to α-glycosylcholine (α-GC) promoted activation of invariant natural killer (iNK) cells and enhanced CD4^+^ T cell responses.

**Figure 8:**
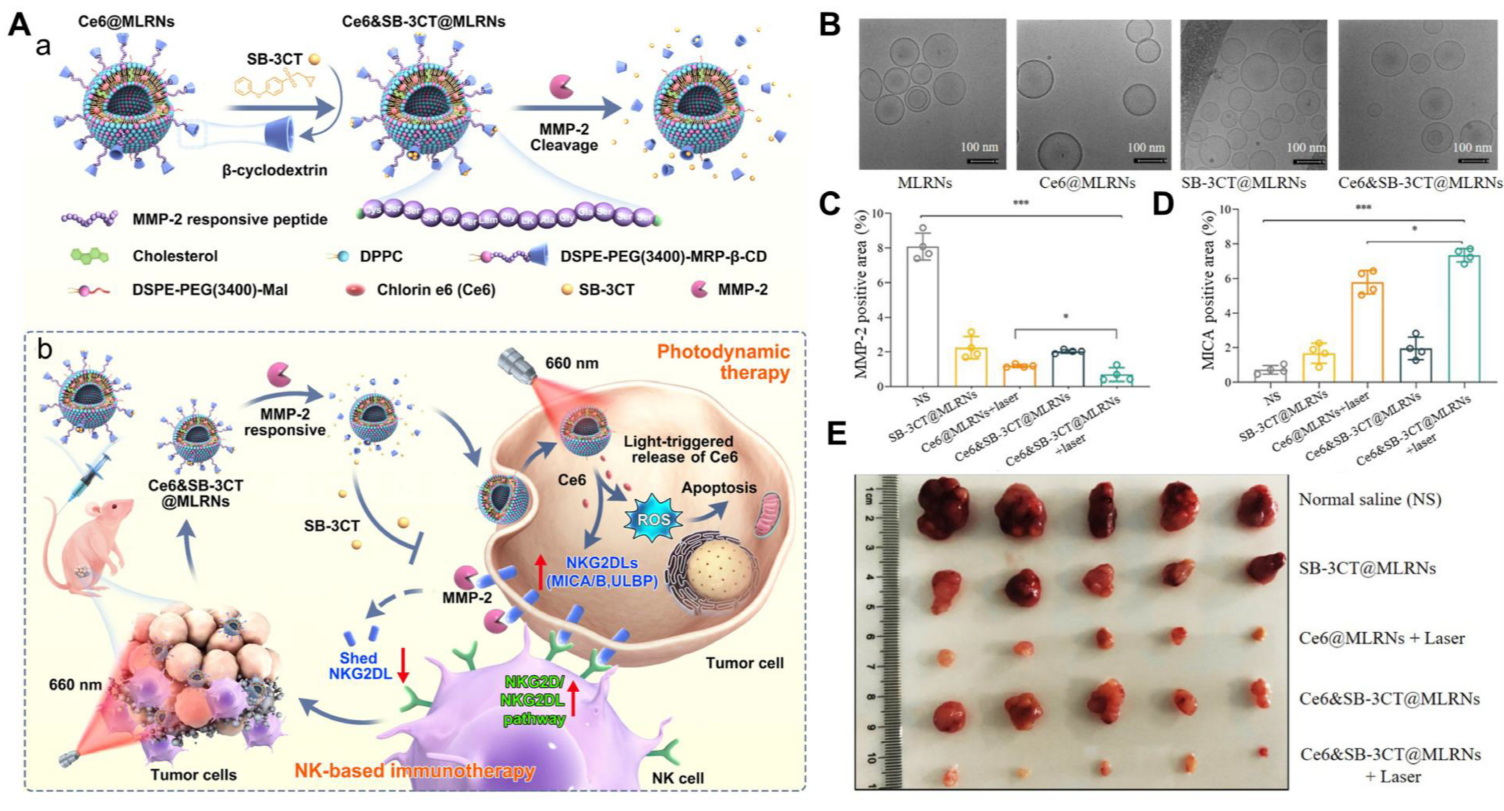
Peptide/glycans dual-modified liposomal drug delivery systems for cancer photo-immunotherapy. (A) (a) Schematic representation of the synthetic route for the preparation of Ce6&SB-3CT@MLRNs. (b) Illustration of the dual-responsive drug release behavior and the underlying mechanism by which Ce6&SB-3CT@MLRNs enhance photodynamic immunotherapy in tumors. (B) Transmission electron microscopy (TEM) images of different MLRN formulations, including blank MLRNs, Ce6@MLRNs loaded with Ce6 alone, SB-3CT@MLRNs loaded with SB-3CT alone, and Ce6&SB-3CT@MLRNs co-loaded with both Ce6 and SB-3CT. (C, D) Quantitative analysis of the mean positive area percentages of MMP-2 and MICA. *P < 0.05, ***P < 0.001. (E) Photographs of the tumor tissues of mice from different treatment groups. Adapted with permission from 171, Copyright 2023, American Chemical Society.

Attractively, multifunctional liposomal drug delivery platforms based on cyclodextrin and matrix metalloproteinase-2 (MMP-2)-responsive peptides can enable effective cancer photo-immunotherapy. Cyclodextrin (CD) has been used in drug delivery since the 1950s. CDs possess a mildly hydrophobic central cavity that can encapsulate lipophilic drugs or drug molecules. The formation of CD complexes enhances drug solubility, improves chemical stability, and reduces irritation 165, 166. With advancements in biotechnological manufacturing, CD has achieved large-scale production and increased interest in the pharmaceutical industry. Over the past decade, CD has been primarily used in drug delivery systems to enhance the solubility and stability of small-molecule drugs 167. Owing to these properties, CD-based drug delivery systems have demonstrated improved bioavailability and therapeutic efficacy in numerous clinical studies. Interestingly, β-cyclodextrin (β-CD) is a cyclodextrin with an alpha-(1->4) linkage and contains seven D-glucopyranose units 168, 169. Some clinical trials have investigated the tumor-targeting properties of β-CD. For example, a CD dimer synthesized via click chemistry connected hydrophobic and hydrophilic portions, resulting in a self-assembled, noncovalently bonded micellar nanostructure. The researchers validated the fabrication strategy for the drug-loaded nanocarrier and demonstrated its potential for tumor-targeted therapy through endocytosis-related studies 170. More importantly, β-CD-based liposomal drug delivery systems can achieve precise tumor targeting and effective cancer combination therapy. Although PDT combined with immunotherapy has been extensively studied, research has primarily focused on inducing ICD to activate CTLs. In contrast, leveraging the synergistic effects of PDT and NK cell immunotherapy, Liu *et al*. 171 designed a TME/light dual-responsive liposome system (MLRN) to achieve Ce6-mediated PDT combined with NKG2D-enhanced immunotherapy using the therapeutic agent SB-3CT (**Figure 8A**). The system (Ce6&SB-3CT@MLRNs) comprised two components: SB-3CT-loaded β-CD and Ce6-loaded nanoliposomes, connected via an MMP-2-responsive peptide (**Figure 8B**). SB-3CT is a potent and selective competitive inhibitor of MMP-2 and MMP-9, with significant anticancer activity. In the tumor microenvironment, abundant MMP-2 triggered β-CD cleavage and subsequent SB-3CT release (**Figure 8C, D**). After the nanoplatform accumulated within melanoma tissues, the liberated SB-3CT inhibited MMP-2 activity, thereby reducing the shedding of soluble NKG2D ligands and increasing their retention on the tumor cell surface. Simultaneously, Ce6-loaded liposomes were activated by 660 nm laser irradiation, inducing apoptotic death of tumor cells. Through this spatiotemporally coordinated photoimmunotherapeutic strategy, the liposomal system markedly promoted cytotoxicity against A375 melanoma cells, reaching 83.31%, suppressed tumor progression, with a tumor proliferation rate of only 1.13% relative to the saline control, and increased NK cell abundance among tumor-infiltrating lymphocytes (**Figure 8E**).

CDT is a novel therapeutic strategy that selectively kills tumor cells by activating TME-specific Fenton (or Fenton-like) reactions to generate hydroxyl radicals (·OH). CDT primarily relies on nanocatalysts to exploit the elevated H₂O₂ levels within the tumor microenvironment, converting them into highly toxic reactive oxygen species. This process amplifies oxidative stress in malignant cells, causes severe intracellular damage, and ultimately promotes tumor cell death 172-174. Compared to traditional therapies, CDT requires no external excitation and exhibits higher specificity. Notably, CDT can be combined with chemotherapy and magnetic resonance imaging (MRI) to enhance tumor-killing efficacy. Manganese dioxide (MnO₂), a typical CDT agent, reacts with endogenous H₂O₂ in the tumor microenvironment to generate oxygen. The oxygen released from subsequent reactions alleviates the hypoxic TME, attenuates HIF-1α expression, and ultimately reverses multidrug-resistant (MDR) tumors 175, 176. In addition, MnO₂ can be reduced to Mn²⁺ ions at lower pH values in the acidic TME, making it suitable for MRI 177, 178. Therefore, drug delivery systems integrating CDT, chemotherapy, and MRI are extensively researched. For example, Liang *et al*. 179 engineered a dual-ligand-functionalized liposomal platform incorporating NAG/R8 modification and stimuli-responsive dePEGylation. This system was co-loaded with MnO₂ and paclitaxel to enable MRI-guided synergistic chemotherapy and chemodynamic therapy for lung cancer. The active targeting molecule *N*-acetyl-d-glucosamine (NAG), a monosaccharide derivative of glucose, simultaneously targets GLUT1 and lectin receptors. The synergistic interaction of PEG, NAG, and R8 enhanced endocytosis. The multistage liposome (C-NAG-R8-PTXL/MnO₂-lip) demonstrated superior performance under hypoxic conditions and effectively reversed hypoxia-induced chemoresistance. Concurrently, O₂-induced liposome disruption significantly promoted PTX release. The reaction between H₂O₂ and MnO₂ generated highly toxic •OH radicals, which acted synergistically with PTX to exert anti-NSCLC effects. Furthermore, *in vivo* experiments showed that C-NAG-R8-PTXL/MnO₂-lip exhibited outstanding T1-weighted imaging performance.

#### 5.5.2. Dual aptamer

Based on two types of DNA aptamers (anti-CD44 and anti-PD-L1), Kim and colleagues 180 a nanosized cationic liposomal system, Aptm [DOX/IDO1], loaded with DOX and IDO1 siRNA for targeted chemoimmunotherapy and TME modulation. This nanoliposome effectively delivered DOX and IDO1 siRNA via aptamer-mediated endocytosis, successfully binding to breast cancer cells overexpressing CD44 and PD-L1. Among these, DOX was employed as the ICD inducer to kill cancer cells and initiate an anticancer immune response by activating CTLs. Meanwhile, IDO1 siRNA was combined to achieve a synergistic effect by inhibiting DOX-induced IDO1 overexpression. Aptm [DOX/IDO1] promoted the ICD response while reversing the immunosuppressive TME and reducing IDO1 expression in 4T1 tumor-bearing mice, thereby diminishing tumor volume and achieving a highly synergistic antitumor effect. More intriguingly, compared to the saline group, the lungs of Aptm [DOX/IDO1]-treated mice showed no metastatic tumors, exhibiting tissue characteristics similar to normal lungs. These findings indicated that this liposome eliminated breast cancer cells through targeted drug delivery while suppressing tumor metastasis. Collectively, this study developed a tumor-specific multithreaded chemo-immunomodulatory liposomal nanomedicine that enhances chemo-immunotherapy for breast cancer. **Table 3** summarizes various ligand-modified liposomal drug delivery systems for combination cancer therapy.

## 6. Challenges and perspectives

This review systematically summarizes recent advances in ligand-modified liposomal drug delivery systems (LLDDS) for combination cancer therapy. LLDDS creates a potent platform for active targeted drug administration by deftly fusing the inherent benefits of liposomes with the high specificity of ligands for tumor-associated indicators. These liposomes allow for more effective drug accumulation at tumor locations while lowering off-target toxicity, in contrast to traditional passive targeting techniques that mainly depend on the EPR effect. Many studies on various kinds of cancer cells *in vitro* and *in vivo* have shown that LLDDs can increase the therapeutic effect of many drugs, such as traditional chemotherapy and new nucleic acid drugs. The above devices can address the deficiencies of conventional chemotherapy and prolong the time needed for treatment. Ligand modification can also be used to enhance the multi-functionality of liposomes as a drug delivery system. Many problems still need to be solved before the therapeutic use of ligand-modified liposomes can be realised, although some progress has been made.

### 6.1. The shortcomings of ligand-functionalized liposome technology and overcoming strategies

#### 6.1.1. The stability of ligand-functionalized liposomes

First, the addition of functional ligands may reduce the physicochemical stability of liposomes. The above structural modifications may promote phase separation or aggregation in a physiological environment and thus increase the risk of early drug leakage, off-target release and damage to normal tissues. Therefore, to design and optimise ligand-modified liposomal formulations rationally, it is necessary to understand how the surface ligands interact with encapsulated cargo and lipid bilayers. To improve the stability of liposomes, the concentration, kind and spatial distribution of ligands need to be controlled precisely to achieve a uniform arrangement in the lipid bilayer 181. Other factors will also change the circulation time, biodistribution, tumour-targeting efficiency and clearance of these particles, such as particle size and surface charge 182. Among them, one of the problems in developing active targeting nanoparticles is how to set the optimal density for surface ligands on the nanoparticles 183. Although a large number of ligands is expected to bind to the target cells with high affinity, an extremely high density of ligands can also have many negative effects 184. The above reasons lead to a larger nanoparticle size, an increased risk of opsonisation and excessive depletion of receptors on the surface of target cells. Finally, the individual liposomes compete for cellular uptake and thus have reduced targeting efficiency 185. In addition, based on experimental analysis and theoretical calculations, molecular dynamics simulations, differential scanning calorimetry, fluorescence microscopy, etc., can be used to investigate the mechanism of action for the design and optimisation of liposomal carriers to improve their overall performance.

#### 6.1.2. Regulation of protein corona

In addition, studies have shown that ligand modification may lead to the formation of a protein corona (PC) 186. Upon entering the bloodstream, modified liposomes interact with plasma proteins, leading to the formation of a 50-80 nm thick PC layer on their surface. Compared with passively targeted liposomes, the surface molecules and ligands on actively targeted liposomes may promote greater plasma protein binding. The PC influences the circulation time, retention, zeta potential, drug release behavior, and cellular uptake of liposomal delivery systems. More importantly, the PC may also mask ligand-binding sites, thereby reducing targeting efficiency 187. Currently, two main strategies are used to minimize PC formation. The first approach is to use PEG-modified liposomes. The second approach is to use diphosphate polymers as materials for constructing liposomal formulations. A key feature of these materials is their electrostatic interaction with water molecules, which enables the formation of a hydration layer and thereby reduces nonspecific protein adsorption. Interestingly, AI technologies, including machine learning (ML) and deep learning (DL), have shown considerable potential in early cancer diagnosis and the systematic design of nanoplatforms 188. Simultaneously, based on published studies, AI can be used to predict the possible composition of the PC and its biological effects *in vivo* 189, 190.

#### 6.1.3. Integration of stimulus-responsive strategies

Currently, most ligand-modified liposomes still rely on passive drug release, which limits their ability to achieve precise and controlled drug delivery *in vivo*. Passive drug release can lead to premature drug leakage, insufficient therapeutic concentrations at target sites, and toxicity in normal tissues, thereby compromising both efficacy and safety. Endogenous stimulus-responsive strategies can take advantage of tumor-associated factors, such as elevated glutathione levels, overexpressed enzymes, and acidic pH 191. Representative examples include pH-responsive liposomes that undergo structural transformation in acidic microenvironments, as well as enzyme-cleavable linker systems that enable payload release upon exposure to specific proteases. However, these strategies remain insufficiently explored. When designing ligand-modified stimulus-responsive liposomes, exterior stimuli including light, heat, magnetic fields, and ultrasound may be taken into account in addition to internal stimuli.

### 6.2. LLDDS design driven by multiligand modification and imaging technologies

To increase the binding avidity of ligands to their corresponding receptors on target cells, multiple targeting ligands are required to bind to multiple targets on the same cell simultaneously. Single-ligand-functionalised liposomes are often limited by slow receptor turnover and recycling kinetics, thus reducing their efficiency of cellular uptake. Dual-ligand-modified liposomal systems can bind to two different receptor populations simultaneously and thus enhance receptor-mediated endocytosis and improve intracellular delivery. With the continuous development of research in this area, future designs of ligand-modified actively targeted liposomes will increasingly be dual- or even multi-ligand modified. In addition, the design of imaging-technology-driven ligand-modified liposomal drug delivery systems can be realised via various means, such as magnetic resonance imaging (MRI)-guided systems, fluorescence imaging, positron emission tomography (PET)-guided systems, photothermal therapy (PTT)-guided systems, etc., for real-time monitoring of drug uptake and distribution in personalised medicine 192, 193.

### 6.3. Clinical translation

In addition to verifying the excellent anti-tumour effect of ligand-modified liposomal systems, clinical application also requires the development of large-scale, reproducible and economical manufacturing platforms. Functionalized liposomes with specific ligand designs for quality control are a more difficult problem than traditional liposomes. Although some ligands, such as aptamers, have shown safety in early human trials 194, additional steps are still required before they can be included in complex liposomal nanoplatforms. These are the processes of nonclinical safety, clinical comparability, chemistry, production and controls. The above reasons make it more challenging to ensure the consistency of properties for nanomedicines. The U.S.A. At present, the FDA requires assessments of human pharmacokinetics, toxicology and pharmacodynamics for liposomal products and has requested strict control over key quality attributes. Functionalised liposomes are becoming more complex, and their production costs are also rising. For instance, in chemical synthesis of functional lipids and formulation of surface-modification reagents, production costs have risen, quality control has been complicated, and batch-to-batch variability may have occurred; therefore, regulatory approval has become more difficult. To solve the problems above, reformulate and adjust the proportion of basic lipids and functional molecules for better cost performance. Cost-effective manufacturing may also be promoted by using synthetic phospholipids, such as DSPC, DPPC and DOPC, and a modular formulation strategy with simplified surface-functionalisation methods. Build a scalable and highly reproducible production platform for microfluidic-based fabrication and continuous extrusion technology to increase manufacturing efficiency, improve batch-to-batch consistency, and promote broad clinical applications.

## 7. Summary

In short, although ligand-modified liposomal drug delivery systems (LLDDS) are still in the exploration stage, their excellent active targeting ability, compatibility with new therapeutic modes and high biocompatibility make them a promising platform for clinical application. With the continuous progress of target discovery, formulation optimisation, biosafety assessment and large-scale production, LLDDS are expected to show good results.

## Figures and Tables

**Table 1:** The advantages and limitations of each generation of liposomes.

Each generation of liposomes	Advantages	Limitations
Generation I: Ordinary liposome	Co-encapsulation of hydrophilic and hydrophobic drugs; Biocompatibility	Instability; Short half-life of blood; Low drug loading capacity
Generation II: PEG-liposome	Extended blood circulation; Immune evasion	Limited targeting specificity; Reduced drug uptake; Impaired endosomal escape
Generation III: LLDDS	Strong active targeting capability; Enhanced cellular uptake; Minimizing off-target effects; Integrating stimulus-responsive components	Restricted large-scale production

**Table 2:** Common ligands for functionalized liposome modification, corresponding receptors, and typical target cells.

Ligand type	Ligand name	Receptor name	Typical target cells
Peptide	cRGD	integrin αvβ3	Glioblastoma (U87MG), Breast cancer (MDA-MB-231)
	iRGD	integrin αvβ3/αvβ5, NRP-1	Pancreatic cancer (PANC-1)
	LyP-1	p32	Triple negative breast cancer (4T1)
	RVG29	nAChR	Glioma (C6)
	SP94	GRP78	Liver cancer (HepG2)
	GE11	EGFR	Hepatocellular carcinoma (SMMC-7721)
	T7 (HAIYPRH)	TfR1/CD71	Lung cancer (A549)
	T_10_	TfR	Colorectal cancer (HCT-116)
	Octaarginine (R8)	syndecan-4	Broad-spectrum tumor cells (HeLa, A549)
Glycans	Mannose	CD206, MRC1	Dendritic cell, Tumor associated macrophages
	Dextran sulfate	SRA, MSR1, CD204	Tumor associated macrophages
	Fucose	MRC1/CD206	Dendritic cell, Tumor associated macrophages
	Chondroitin sulfate	CD44	Triple negative breast cancer (4T1)
	Hyaluronic acid	CD44	Lung cancer (A549), Ovarian cancer (SKOV-3)
	Galactose	ASGPR	Liver cancer (HepG2)
	GalNAc	ASGPR, ASGR1	Hepatocytes
Aptamer	AS1411	Nucleolin	Breast cancer (MCF-7), Pancreatic cancer (MIA PaCa-2), Glioblastoma (U87MG)
	PtdSer	TIM-4	Macrophage, Dendritic cell
	A10	PSMA	Prostate cancer (LNCaP)
	sgc8	PTK7	Leukemia (CCRF-CEM)
	EpCAM aptamer	EpCAM	Colorectal cancer (CT-26)
Others	Folic acid	FRα, FOLR1	Ovarian cancer (SKOV-3), Cervical cancer (KB)
	Transferrin	TfR1, CD71	Glioblastoma (U87MG), Breast cancer (MCF-7), Leukemia (K562)
	Anti-DC-SIGN nanobody	DC-SIGN/CD209	Immature/monocyte-derived dendritic cells, Certain macrophages
	Anti-CD169 nanobody	CD169, Siglec-1	CD169 positive antigen-presenting cells, Macrophage

**Table 3:** Ligand-modified liposomal drug delivery systems for combination cancer therapy.

Ligand type	Delivery platform	Cargo	Model cancer cell	Combination therapy strategy	Refs.
Peptide	FMSN(BBR)-CXCR4BPL(PTX)	BBR, PTX	Mice colon cancer CT-26 cells	Chemotherapy, Immunotherapy	54
	T_10_/TPZ@M/IR@L	TPZ, IR820	Mice breast cancer 4T1 cells	Chemotherapy, PDT	67
	PoIC/OVA-R8L	OVA	Mice T-cell lymphoma cells	Immunotherapy, PDT	56
	cRGD-PaNPs-αPD-L1	Pa, αPD-L1	Mice breast cancer 4T1 cells	Immunotherapy, PDT	70
	SP65-lipo-CH	CH223191	Mice colon cancer MC38 cells	Chemotherapy, Immunotherapy	53
	C/J-Lipo^RGD^	JQ1, CAT	Mice skin melanoma B16F10 cells	Radiotherapy, Immunotherapy	77
	Lip@inS3-TEG-VL-TK	PROTACs	Mice liver cancer Hepa 1-6-Luc cells	Chemotherapy, Immunotherapy	52
Glycans	PMRL	R848	Mice breast cancer 4T1 cells	Immunotherapy, PTT	98
	GMCP-Lip	CBD, PPD	Mice breast cancer 4T1 cells	Chemotherapy, Immunotherapy	100
	DAPC-Fuc/CpG	CpG ODNs	Mice breast cancer 4T1 cells	Ferroptosis, Immunotherapy	114
Aptamer	MC@RL/Apt	Mn-Ce6	Mice colon cancer CT-26 cells, Mice skin melanoma B16F10 cells	Immunotherapy, PDT	133
	AS1411/lip/PPa/C-R	CDs-RTA conjugates, PPa	Human breast cancer MCF-7 cells	Chemotherapy, PDT	125
	PTX/PS-Zn@Lip-Apt	PTX, AIE PSs, Zn^2+^	Human prostate cancer PC3 cells	Chemotherapy, PDT	126
Folic acid	FPGL, FPCL@pIL-15	pIL-15, GEM	Mice breast cancer 4T1 cells	Chemotherapy, Immunotherapy	142
	Dox-FA-PoP	DOX	Human oral epidermoid carcinoma KB cells	Chemotherapy, PDT	143
Transferrin	TDPL	DHA, PIP	Mice breast cancer 4T1 cells	Ferroptosis, Immunotherapy	149
Nanobody	Nbs-Lip	OVA	Mice breast cancer 4T1 cells	Immunotherapy, PDT	156
Peptide/Glycans	C-NAG-R8-PTXL/MnO2-lip	PTX, MnO_2_	Human non-small cell lung cancer A549 cells	Chemotherapy, CDT, MRI	179
	Ce6&SB-3CT@MLRNs	SB-3CT, Ce6	Human melanoma A375 cells	Immunotherapy, PDT	171
	SLP-αGC-Le^Y^	αGC	Mice melanoma B16 cells	Chemotherapy, Immunotherapy	164
	SBA-pH-Lip	SBA	Mice T-cell lymphoma cells	Immunotherapy, PTT	163
CD44/PD-L1 Aptamer	Aptm [DOX/IDO1]	DOX, IDO1 siRNA	Human breast cancer MDA-MB-231 cells, Mice breast cancer 4T1 cells	Chemotherapy, Immunotherapy	180

## Abbreviations

LLDDS: ligand-modified liposomal drug delivery systems
CAR-T: chimeric antigen receptor T-cell
ICB: immune checkpoint blockade
TDDS: targeted drug delivery systems
EPR: enhanced permeability and retention
NPs: nanoparticles
PTT: photothermal therapy
PDT: photodynamic therapy
CDT: chemodynamic therapy
PEG: polyethylene glycol
MPS: mononuclear phagocyte system
Doxil®: doxorubicin liposome
CPPs: cell-penetrating peptides
CTPs: cell-targeting peptides
ICD: immunogenic cell death
DAMPs: damage-associated molecular patterns
CRT: calreticulin
ATP: adenosine triphosphate
HMGB1: high-mobility group box protein 1
DCs: dendritic cells
TME: tumor microenvironment
STAT3: signal transducer and activator of transcription 3
PROTACs: proteolysis-targeting chimeras
HCC: hepatocellular carcinoma
CSCs: cancer stem cells
AhR: aryl hydrocarbon receptor
IL-12: interleukin-12
IL-6: interleukin-6
IL-10: interleukin-10
IL-15: interleukin-15
TGF-β: transforming growth factor-β
SP65: synthetic peptide 65
DOPE: dioleoylphosphatidylethanolamine
IFN-γ: interferon-γ
TNF-α: tumor necrosis factor-α
AI: artificial intelligence
BBR: berberine
FMSN: functionalized mesoporous silica nanoparticles
PTX: paclitaxel
OVA: ovalbumin
ROS: reactive oxygen species
IL-4: interleukin-4
PC: protein corona
Tf: transferrin
TPZ: tirapazamine
MSNs: mesoporous silica nanoparticles
FDA: Food and Drug Administration
Pa: pheophorbide A
PD-L1: programmed cell death ligand 1
RT: radiotherapy
Man: mannose
MR: mannose receptor
TAAs: tumor-associated antigens
NIR: near-infrared
PDA: polydopamine
GLUT: glucose transporters
AIE: aggregation-induced emission
PCI: photochemical internalization
PtdSer: phosphatidylserine
FRs: folate receptors
GEM: gemcitabine
NKG2DLs: natural killer group 2 member D ligands
Dox: doxorubicin
PIP: piperine
APCs: antigen-presenting cells
CS: chondroitin sulfate
CD44: cluster of differentiation 44
TNBC: triple-negative breast cancer
α-GC: α-glycosylcholine
iNK: invariant natural killer
MMP-2: matrix metalloproteinase-2
β-CD: β-cyclodextrin
MRI: magnetic resonance imaging
MDR: multidrug-resistant
NSCLC: non-small-cell lung cancer
ML: machine learning
DL: deep learning
